# Characterisation of phenotypic patterns in equine exercise‐associated myopathies

**DOI:** 10.1111/evj.14128

**Published:** 2024-07-05

**Authors:** Victoria Lindsay‐McGee, Claire Massey, Ying Ting Li, Emily L. Clark, Androniki Psifidi, Richard J. Piercy

**Affiliations:** ^1^ Department of Clinical Sciences and Services Royal Veterinary College London UK; ^2^ The Roslin Institute, University of Edinburgh Edinburgh UK; ^3^ Present address: Royal (Dick) School of Veterinary Studies University of Edinburgh Edinburgh UK

**Keywords:** disease subtypes, EAMS, exercise‐associated myopathy syndrome, exertional rhabdomyolysis, myopathy, RER

## Abstract

**Background:**

Equine exercise‐associated myopathies are prevalent, clinically heterogeneous, generally idiopathic disorders characterised by episodes of myofibre damage that occur in association with exercise. Episodes are intermittent and vary within and between affected horses and across breeds. The aetiopathogenesis is often unclear; there might be multiple causes. Poor phenotypic characterisation hinders genetic and other disease analyses.

**Objectives:**

The aim of this study was to characterise phenotypic patterns across exercise‐associated myopathies in horses.

**Study design:**

Historical cross‐sectional study, with subsequent masked case–control validation study.

**Methods:**

Historical clinical and histological features from muscle samples (*n* = 109) were used for *k*‐means clustering and validated using principal components analysis and hierarchical clustering. For further validation, a blinded histological study (69 horses) was conducted comparing two phenotypic groups with selected controls and horses with histopathological features characterised by myofibrillar disruption.

**Results:**

We identified two distinct broad phenotypes: a non‐classic exercise‐associated myopathy syndrome (EAMS) subtype was associated with practitioner‐described signs of apparent muscle pain (*p* < 0.001), reluctance to move (10.85, *p* = 0.001), abnormal gait (*p* < 0.001), ataxia (*p* = 0.001) and paresis (*p* = 0.001); while a non‐specific classic RER subtype was not uniquely associated with any particular variables. No histological differences were identified between subtypes in the validation study, and no identifying histopathological features for other equine myopathies identified in either subtype.

**Main limitations:**

Lack of an independent validation population; small sample size of smaller identified subtypes; lack of positive control myofibrillar myopathy cases; case descriptions derived from multiple independent and unblinded practitioners.

**Conclusions:**

This is the first study using computational clustering methods to identify phenotypic patterns in equine exercise‐associated myopathies, and suggests that differences in patterns of presenting clinical signs support multiple disease subtypes, with EAMS a novel subtype not previously described. Routine muscle histopathology was not helpful in sub‐categorising the phenotypes in our population.

## Résumé

**Contexte:**

Les myopathies induites à l'exercice demeurent fréquentes, hétérogènes cliniquement et représentent des désordres idiopathiques caractérisés par des épisodes de dommages myofibrillaires en lien avec l'exercice. Les épisodes sont intermittents et varient à la fois chez le même cheval, entre chevaux et entre les différentes races. L'étiopathogénie demeure obscure et pourrait être multifactorielle. La pauvre caractérisation phénotypique des myopathies ne simplifie pas les analyses génétiques ni celles d'autres maladies.

**Objectifs:**

Le but de cette étude est de caractériser les patrons phénotypiques en lien avec les myopathies induites à l'exercice chez le cheval.

**Type d'étude:**

Étude transversale historique et étude subséquente de validation de cas témoins aveugle.

**Méthodes:**

Les facteurs clés cliniques et histologiques provenant d'échantillons de muscles (n = 109) ont été utilisés pour l'algorithme de K‐moyennes et validés par le biais d'analyse des composantes principales et de classification hiérarchique. Pour validation additionnelle, une étude histologique à l'aveugle (69 chevaux) a été faite comparant les deux groupes phénotypiques avec des contrôles sélectionnés et des chevaux avec éléments histopathologiques caractérisés par de la discontinuité myofibrillaire.

**Résultats:**

Deux phénotypes distincts ont été identifiés: un premier sous‐type de syndrome de myopathie induite à l'exercice non‐classique (EAMS) associé à de la douleur musculaire telle que décrite par le praticien suivant le cheval (χ^2^_(df=1,n=109)_ = 19.33, p < 0.001), difficulté à se déplacer (χ^2^_(df=1,n=109)_ = 10.85, p = 0.001), démarche anormale (χ^2^_(df=1,n=109)_ = 34.61, p < 0.001), ataxie (χ^2^_(df=1,n=109)_ = 10.88, p = 0.001) et parésie (χ^2^_(df=1,n=109)_ = 10.88, p = 0.001); alors qu'un sous‐type RER classique non‐spécifique n'était associé à aucune variable en particulier. Aucune différente histologique n'a été identifié entre les sous‐types dans l'étude de validation et aucune caractéristique histopathologique d'autres myopathies équines n'a été identifiées dans les différents sous‐types.

**Limites principales:**

Aucune population indépendante pour validation; petite taille d'échantillon pour les sous‐types peu nombreux identifiés; aucun cas contrôles positifs de myopathie fibrillaire; description des cas provenant de multiples praticiens indépendants et non‐aveugles.

**Conclusion:**

Cette étude est la première utilisant des méthodes de regroupement informatique pour identifier des patrons phénotypiques de myopathies équines induites à l'exercice et suggère que des différences existent dans les patrons de signes cliniques en faveur de multiples sous‐types de maladie, incluant EAMS qui représente un nouveau sous‐type non décrit jusqu'à maintenant. L'histopathologie musculaire de routine n'a pas permis de sous‐catégoriser les phénotypes dans cette population.

## 1 INTRODUCTION

Given that horses are commonly used for athletic pursuits, it is perhaps unsurprising that exercise‐associated muscle disorders are commonly recognised in this species. Often, affected horses are presumptively diagnosed with exertional rhabdomyolysis (ER), a clinically heterogeneous myopathic syndrome characterised by exercise‐induced episodes of myofibre damage. Clinical signs of ER can include stiffness, muscle fasciculation, cramping, sweating, reluctance to move, myoglobinuria, and in severe cases recumbency, or death. Typically, the clinical presentations and severities of ER vary between both individuals and between episodes. ER is a rare disease in humans, where highly active people such as military personnel^1^, ^2^, ^3^, ^4^ and athletes^5^, ^6^, ^7^, ^8^, ^9^ are commonly affected. However, ER is much more common in equivalently athletic horses: approximately 5%–7% of racing Thoroughbreds are affected.^10^, ^11^, ^12^ While some horses can have a spontaneous single episode of ER, many horses have recurring episodes: a syndrome known as recurrent ER (RER). Together with the welfare implications, this syndrome also has a substantial financial impact. One study on racehorses revealed that six training days were lost per episode, and 68% of horses were prevented from racing during the previous year.^11^ However, beyond the racing industry, a variety of different breeds and a range of other disciplines such as harness racing,^13^ polo,^14^ eventing^15^ and endurance riding^16^ are also affected by exercise‐associated myopathies. RER is a heritable condition in both Thoroughbreds (*h* ^2^ = 0.34–0.46) and Standardbreds (*h* ^2^ = 0.39–0.49),^17^ but genome‐wide association studies have, so far, not found a putative causal genetic variant associated with RER.^18^, ^19^ Similarities in disease presentation has led to assumptions that the aetiology is similar or identical between and within breeds; however, without definitive testing, this remains an assumption. Differences in presentation might be overlooked in individual cases—differences that otherwise might help define distinct subtypes.

RER syndrome is non‐specific and largely idiopathic—the syndrome is generally characterised by repeated exercise‐associated ER episodes of muscle stiffness or pain, and variable and intermittent post‐episode elevations in muscle‐derived enzyme activities. RER is also histologically non‐specific, associated with evidence of myofibre damage and regeneration typical of many myopathies (i.e., presence of internalised myonuclei or myofibre size variation), but no RER‐specific associated histological features. Diagnosis is often made based on exercise‐association of clinical signs and on exclusion of other exercise‐related myopathies such as type 1 polysaccharide storage myopathy (PSSM). Previous attempts to classify^20^ equine RER have instead focused on the apparent triggering factors of ER episodes. In contrast, human ER is associated with a range of distinct genetic diseases, including glycogen metabolism disorders, long‐chain fatty acid metabolism disorders, mitochondrial disorders, calcium influx disorders, caveolinopathies, limb girdle muscular dystrophies, sarcoglycanopathies, dystrophinopathies and sickle cell trait.^21^ Human ER is subsequently often treated more as a symptom than a specific disease in its own right. It is possible that RER syndrome in horses could also represent a range of distinct exercise‐associated myopathies or disease subtypes, particularly in different (especially unrelated) breeds.

There is precedent for identifying novel myopathies within RER syndrome in horses. In the early 1990s, Valberg et al.^22^ identified a subpopulation of Quarter Horses (QH) with RER that displayed a distinct histological phenotype: periodic Acid‐Schiff (PAS)‐positive inclusions in type 2 muscle fibres, identified as an amylase‐resistant abnormal polysaccharide (polyglucosan). Various other differences in the clinical and histological phenotype of these horses with this newly identified PSSM when compared with horses with other forms of ER were identified, and additional breeds were reported with the same disease based on histopathological criteria.^23^, ^24^, ^25^, ^26^, ^27^, ^28^, ^29^, ^30^, ^31^ Then, in 2008 a p.R309H mutation in the glycogen synthase (*GYS1*) gene was identified^32^ and the gain of function disease mechanism subsequently explained.^33^

Not all horses with a PSSM histological phenotype are positive for the p.R309H mutation.^26^, ^32^, ^34^, ^35^ Consequently, those with the mutation are now designated as PSSM Type 1 (PSSM1), while those without, are designated as PSSM Type 2 (PSSM2). Currently, there is no genetic mutation nor causal disease pathway identified for PSSM2, nor for other ER‐associated diseases, and the possibility remains that either, or both, PSSM2 and RER represent a syndrome comprising multiple diseases, or there is phenotypic heterogeneity (either clinical or histological) leading to phenotypically distinct disease subtypes with differing underlying aetiopathogenesis. More recently, groups of horses of certain breeds (Warmblood^36^ and Arabian^37^) with ER have been reported with a histopathologically distinct myopathy, termed myofibrillar myopathy (MFM), a broad term that in humans encompasses a heterogenous group of inherited disorders with a variety of genetic causes.^38^, ^39^, ^40^, ^41^ These horses have been described as having aggregates of the normal intermediate filament muscle protein, desmin, in occasional myofibres.^36^, ^37^ It remains unclear whether these animals have a separate disease or diseases or whether they contribute to the large and apparent, ill‐defined pool of affected animals. Further, and unfortunately, the confusion surrounding defining RER phenotypes has been exacerbated by uncorroborated, but nonetheless, popular genetic testing for several of these disorders using unsubstantiated assays that currently lack validity.^42^, ^43^

The aim of this study was to test the hypothesis that there are subgroups of exercise‐associated myopathies within horses that can be defined by clinical signs and by histopathological features in muscle biopsy samples. We used historical case records from the RVC's Comparative Neuromuscular Diseases Laboratory biopsy database and following an unbiased computational clustering analysis of the retrospective data to define phenotypes, we then performed a prospective, blinded, reassessment of histopathological features.

## 2 MATERIALS AND METHODS

### 2.1 Study design

**FIGURE 1:**
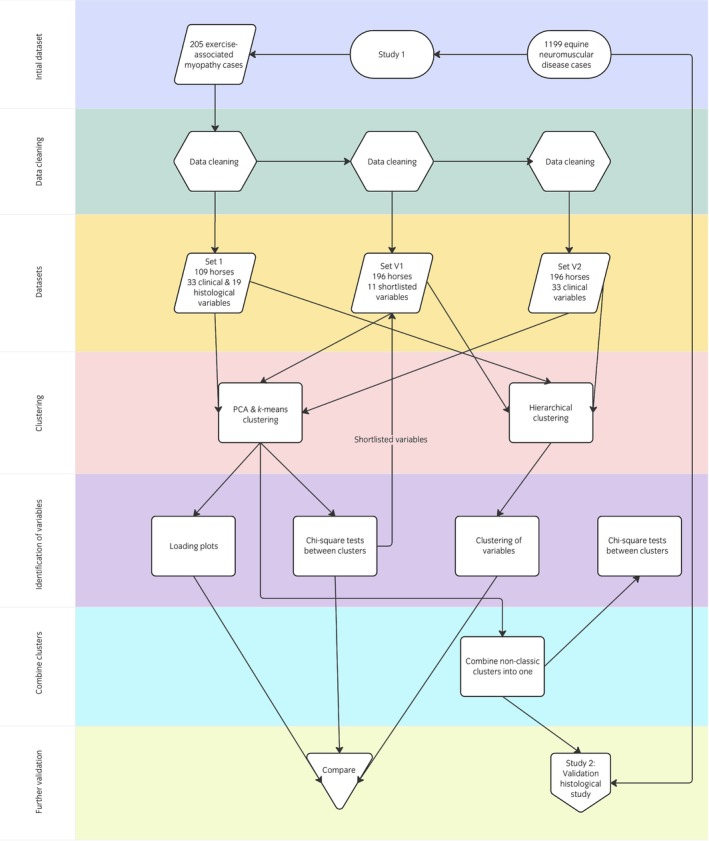
Flowchart (produced using Miro^44^ software) describing the steps in Study 1, including the data curation and cleaning, the cluster analysis and identification of variables associated with distinct clusters, followed by Study 2, the validation histological study.

In this article, we present two linked studies: Study 1, a clustering analysis of retrospective data from clinical samples in order to define phenotypic subtypes; and Study 2, a prospective, blinded validation study. Figure 1 is a flow chart presenting the steps taken in Study 1, leading to Study 2.

Briefly, in Study 1, exercise‐associated myopathy cases were selected from the wider neuromuscular database, and split into three datasets, a primary dataset (Set 1) and two validation datasets (Set V1 and Set V2), each of a larger sample size but fewer variables than the primary dataset, in order to validate the findings in Set 1. These datasets underwent a primary clustering analysis of principal components analysis (PCA) and *k*‐means clustering, then a secondary comparative method of hierarchical clustering. This allowed us to compare between the primary and validation datasets as well as between different computational clustering methods, to better support our findings. Variables significantly associated with the identified clusters were identified and compared. In Study 2, we selected horses from the identified clusters (potential subtypes), and these underwent a prospective, blinded, histological validation study.

### 2.2 Study 1: Phenotype clustering using diagnostic biopsy service records

#### 2.2.1 Data curation and cleaning

Diagnostic reports for 1199 muscle biopsy and blood samples submitted to the RVC's Comparative Neuromuscular Diseases Laboratory (CNMDL) referral service from 2006 to 2017 inclusive were collated for this study. All original contemporaneous diagnoses were made by, and reports written, by a single person (co‐author RP), skilled in interpretation of muscle biopsy histopathology. Signalment data were recorded for each animal, as was clinical history data as reported by the submitting veterinary surgeon, results of genetic testing where applicable, environmental factors, biochemistry and the histological features identified on the muscle biopsy sample (full list of variables (*n* = 172)) (Table S1). All clinical history and histological feature data were logged as binary variables (reported versus not reported in relation to the clinical history, and present versus not present in histological features) except for the histological disease stage (classified as acute, acute‐chronic or chronic), histological severity, degree of nuclei internalisation, increase in fibre size variation (all rated on an ordinal scale from 0 to 5), and biochemistry data such as creatine kinase (CK) and aspartate amino transferase (AST) activities (binned into ordinal scores).

Horses with a diagnosis of ‘idiopathic myopathy’ and reported exercise association were then extracted from the database, and horses with a diagnosis of PSSM, mitochondrial myopathy, vacuolar myopathy or other defined myopathy were excluded, leaving a dataset of 205 that we then referred to as exercise‐associated myopathy syndrome horses. All variables that contained no variation in these horses (such as clinical or histological signs associated with other neuromuscular diseases from the original 1199 horse biobank database, e.g., laryngeal paresis as a defining feature of recurrent laryngeal neuropathy) were dropped, as were variables with more than 50% missing data (this included PSSM1 genetic test results, electromyography and scintigraphy results). Signalment data were then masked for the automated clustering analysis, to avoid clustering based on patterns in signalment rather than disease phenotypes. After removing horses with missing data, 109 horses with both clinical history and histological features remained for further analysis. Finally, a total of 33 clinical history variables and 19 histological feature variables were included (full list of recorded and included variables can be found in Table S1). We termed this dataset Set 1.

The remaining variables were then standardised using *StandardScaler* from the scikitlearn library,^45^ which uses a Z‐scoring normalisation method whereby each variable is transformed so that the mean is 0 and the variance is 1. Then a matrix of Spearman's correlations between variables was produced using *corr* from the pandas library,^46^ and plotted as a heat map using *heatmap* from the seaborn library.^47^

#### 2.2.2 Principal components analysis and *k*‐means clustering

A principal components analysis (PCA) was carried out on the Set 1 standardised clinical history and histological features data using *PCA* from the scikitlearn library^45^ and an elbow plot of variance explained by principal component (PC) produced using *lineplot* from seaborn.^47^ To select the number of centroids for *k*‐means clustering, the sum of squared distances from *k*‐means clustering analyses on the standardised dataset for the range of *k* from 1 to 30 were calculated and plotted as an elbow plot. *K*‐means clustering was then carried out on the standardised data using the optimal identified value for k. The computer‐assigned clusters, termed phenotypic subtypes, were then indicated on PCA score plots using *Pairgrid* from seaborn^47^ and in a three‐dimensional scatter plot using matplotlib.^48^ Loadings were extracted for each variable, and variables with loadings >0.2 or <−0.2 on each of the first three principal components were included on biplots using matplotlib.^48^

To identify the variables that were significantly associated with each cluster identified by the *k*‐means clustering algorithm (phenotypic subtype), firstly the full list of signalment (including age, breed and sex), clinical and histological variables was reduced from 61 to 34 using two feature selection methods to identify the best 25 variables: *SelectKBest* from scikitlearn,^45^ which ranks variables based on Chi statistics; and *ExtraTreesClassifier* from scikitlearn, which runs a random forest algorithm and produces relative feature importance scores based on the normalised total reduction in the mathematical criteria used in the decision of which feature where the tree splits. This reduced the variable list to a longlist of 34. The longlist variables were then tested individually using a Chi‐squared (*χ* ^2^) test to identify important distinguishing variables between the phenotypic subtypes, with a Bonferroni‐corrected significance threshold of *p* < 0.0015, and a nominal *p* < 0.05 considered suggestive (i.e., not statistically significant, but possibly worth future study in a larger cohort).

One phenotypic subtype (labelled the ‘classic RER’ subtype, the definition of which is discussed in more depth in the results) was then compared against the combined other groups using *χ* ^2^ tests on the same variable longlist, and the variables that were significant or suggestive, either between all four phenotypic subtypes or between classic and combined non‐classic (termed exercise‐associated myopathy syndrome or EAMS) subtypes, were used as the shortlisted variables for further study. Spearman's correlations were calculated between shortlisted variables and phenotypic subtypes and plotted as a heat map as above.

Differences in reported serum CK activities were also compared. Due to inconsistency in reporting from first opinion veterinary surgeons, ranging from specific CK activities at varying points post exercise or post episode, to reporting ranges or simply reporting CK activity as ‘normal’, and due to the unavoidable use of different laboratories and practice equipment for measuring CK activity, the data were binned into ordinal scores as detailed in Table S1. CK activity was then compared both between phenotypic subtypes (using Kruskal–Wallis test) and between classic RER and non‐classic EAMS subtypes (two‐sided Mann–Whitney *U*), in 49 horses.

#### 2.2.3 Validation using reduced input variables

To validate the robustness of the automated clustering and increase the number of horses from the initial dataset with an assigned phenotypic subtype, two larger validation datasets were also produced from the original 205 horse dataset: Set V1 (*n* = 196) consisted only of the shortlisted variables from the analysis of Set 1; Set V2 (*n* = 196) consisted of all 33 clinical history variables.

PCA and *k*‐means clustering were run on both sets as described above, and significantly associated clinical signs in Set V2 were identified as described above for Set 1. Differences in serum CK activities were also compared both between phenotypic subtypes (using Kruskal–Wallis test) and between classic and non‐classic EAMS subtypes (two‐sided Mann–Whitney *U* test), in 98 horses.

Percentage agreement between the Set 1 clustering results (using all final histological and clinical history variables in 109 horses) and the Set V1 (using the shortlisted variables) and Set V2 (using the clinical history variables only) clustering was calculated from a contingency table, and the area under the curve (AUC) from the receiver operating characteristic (ROC) curve between the initial clustering results and the two validation sets was also compared. Spearman's correlations between the shortlisted variables and the classic RER and non‐classic EAMS subtypes from each analysis were calculated as above.

#### 2.2.4 Validation using hierarchical clustering

Hierarchical clustering on Set 1 plus the two validation sets (Set V1 and Set V2) was also carried out. Agglomerative clustering using Ward's method^49^ of cluster similarity calculation was applied to the three sets and to the three lists of variables (all histological and clinical history variables, the shortlisted variables, and all clinical history variables, respectively) to assess grouping of variables. The results were then compared against the respective *k*‐means clustering results. This analysis was carried out using *clustermap* from the seaborn library.

### 2.3 Study 2: Histopathological comparison of phenotypic‐based exercise‐associated myopathy subtypes

As the initial reports on submitted referral biopsied muscle sample were written over a 12‐year period and were performed with the observer not blinded to clinical and signalment data, and furthermore, the subjective process of assessment of biopsy samples might have altered over that period, histopathological assessments were blindly repeated and compared between horses within the classic and non‐classic disease subtypes. In addition, due to similarity between some identified significant clinical signs in some of our horses and those Valberg et al. described as associated with equine MFM,^36^, ^37^ a comparison of desmin immunohistochemistry was performed (sarcoplasmic desmin aggregates being the reported defining histopathological feature for MFM^36^, ^37^).

#### 2.3.1 Sample selection

Of the 196 horses from Set V1 assigned a phenotypic subtype, we selected Thoroughbred, Warmblood or Arabian horses (Arabians and Warmbloods described as breeds associated with equine MFM^36^, ^37^) for which semimembranosus muscle biopsy samples were available from the CNMDL referral neuromuscular diagnostic service biobank with owner consent for research use. Due to the nature of the biobanked samples as originating from a diagnostic service for animals with clinical abnormalities, there was a lack of availability of healthy control tissue. Instead, controls were semimembranosus muscle biopsy samples from the original 1199 horse biobank dataset, where horses had no history of exercise‐associated myopathy and unrelated diagnoses based on histology and clinical history: in the majority, these samples had been considered histologically normal. In addition, horses for which the original report described histological features of myofibrillar separation and/or disruption or of myofibrillar aggregates with submitted semimembranosus tissue were combined into a ‘myofibrillar abnormalities’ group. While desmin staining is not carried out routinely as part of the CNMDL diagnostic service and was not included for the original reports for these horses, these cases were identified as horses that otherwise might meet the description of equine MFM and could possibly act as a further positive control comparison group.

Sixty‐nine horses in total were included. A description of all samples included by breed and group are summarised in Table 1.

**TABLE 1:** Samples used for Study 2: Histological comparison of exercise‐associated myopathy subtypes.

Breed	Classic RER	Non‐classic EAMS	Negative control	Myofibrillar defects	Total
Arabian	4	2	1	3	10
Warmblood	13	6	13	2	34
Thoroughbred	7	4	12	2	25
Total	24	12	26	7	69

#### 2.3.2 Histology

Fresh equine muscle biopsy samples submitted by practitioners from the semimembranosus muscle were frozen in isopentane, precooled in liquid nitrogen on cork discs, within 24 h, using standard methods.^50^ They were kept in long term storage at −80°C. Samples were warmed to −22°C in a cryostat (Bright) before sectioning. Other than for PAS (16 μm), all sections were 8 μm in thickness. Using routine methods,^50^ a panel of nine stains was applied to each sample: haematoxylin and eosin (H&E); PAS; amylase pre‐digested PAS; oil red O (ORO); modified Gomori trichrome (TRI); cytochrome oxidase activity (COX); succinate dehydrogenase activity (SDH); nicotinamide adenine dinucleotide tetrazolium reductase activity (NADH); and desmin immunohistochemistry (DES IHC). The latter was conducted routinely, using an anti‐desmin monoclonal mouse antibody, clone D33, Agilent (M0760), diluted 1:50 in phosphate buffered saline (PBS) incubated for 60 min. Biotinylated sheep anti‐mouse IgG secondary antibody, Cytiva (RPN1001), was applied at 1:200 dilution in PBS for 45 min followed by peroxidase‐conjugated streptavidin, Stratech (016‐030‐084‐JIR), diluted 1:500 in PBS for 30 min. Finally 3,3′‐diaminobenzidine (DAB)^50^ Enhanced Liquid Substrate System tetrahydrochloride, Sigma (D3939), diluted according to manufacturer's instructions was applied for 5 min.

#### 2.3.3 Scoring and data analysis

Histological sections were all scored by the same specialist in neuromuscular histopathology, who was blinded to group and horse signalment. The full list of assessed variables is given in Table S3. Briefly, a range of standard muscle histopathological features was ordinally scored for severity, and for distribution (whether diffuse, regional, or focal in distribution throughout the sample). Some free text variables were included, and these were analysed for themes and assigned to dummy variables based on theme. These scores were then cleaned and analysed.

Every variable was subsequently compared across the four groups and between only the two exercise‐associated myopathy groups, either using contingency tables (for categorical variables) or two‐way ANOVA (for ordinal variables) with breed also compared. Variables with a *p*‐value of less than 0.1 were retained and used in a logistic regression model (with exercise‐associated myopathy group as outcome) or multinomial regression model (with disease group as the outcome) and reduced using backward stepwise elimination. This was carried out both when keeping breed dummy variables in the model, and without. These analyses were then repeated while excluding TBs from the dataset, as equine MFM has only been described in WBs and Arabians.^36^, ^37^ Two significance thresholds were used: *p* < 0.05 for statistical significance, and *p* < 0.1 for suggestive variables possibly worth future study but not significant in this analysis.

For PCA, variables with more than 40% missing data were removed, and then all cases with missing data were dropped, leaving 47 cases and 54 variables for further analysis. The 54 variables were normalised using Z‐score normalisation as detailed above, prior to conducting PCA. Loadings were then extracted for each variable, and variables with loadings >0.2 or <−0.2 on each of the first three principal components were included on biplots using matplotlib.

## 3 RESULTS

### 3.1 Phenotype clustering using diagnostic biopsy service records

#### 3.1.1 Principal components analysis and *k*‐means clustering

**FIGURE 2:**
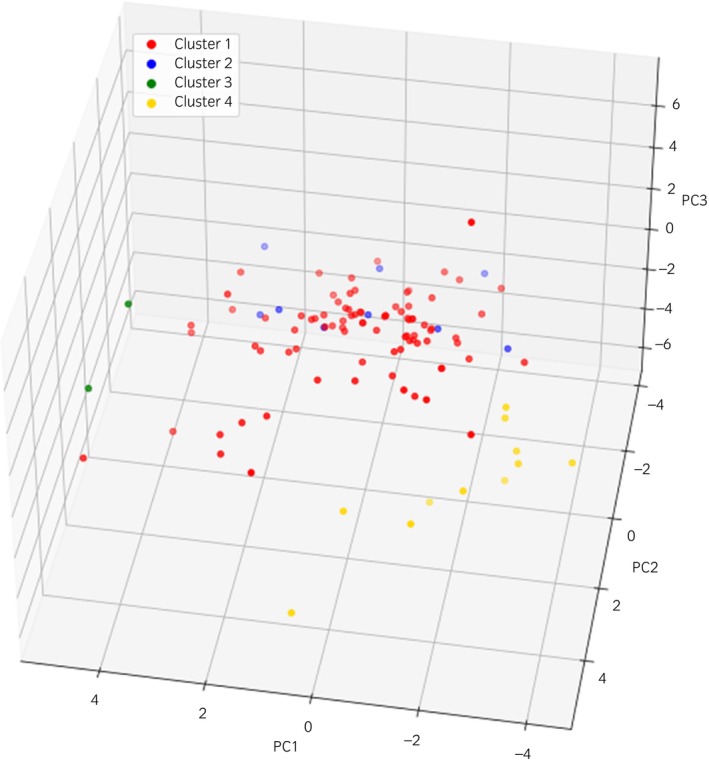
Three‐dimensional scatter plot of the first three principal components (PCs) from the PCA of histological and clinical history data in Set 1 (*n* = 109). Datapoints are coloured by the *k*‐means assigned cluster (phenotypic subtype) assigned to each animal (Clusters 1–4). The first three PCs explained 6.9%, 6.1% and 5.1% of variation in Set 1, respectively. Cluster 1 represented the classic RER phenotype, and Clusters 2–4 the non‐classic EAMS group.

There was no clearly defined elbow in the elbow plot (Figure S1); however, the first point at which the slope of the trend line noticeably decreased, occurred between four and five clusters, so four clusters were selected going forward. Each horse was assigned to a phenotypic subtype using *k*‐means clustering, and this was highlighted on a three‐dimensional plot of the first three principal components (Figure 2). Phenotypic subtype 1 was the largest and most central phenotypic subtype, with PC1 separating out phenotypic subtype 3 and to a lesser extent phenotypic subtype 4, PC2 separating out phenotypic subtypes 2 and 4, and PC3 separating out phenotypic subtype 4.

**FIGURE 3:**
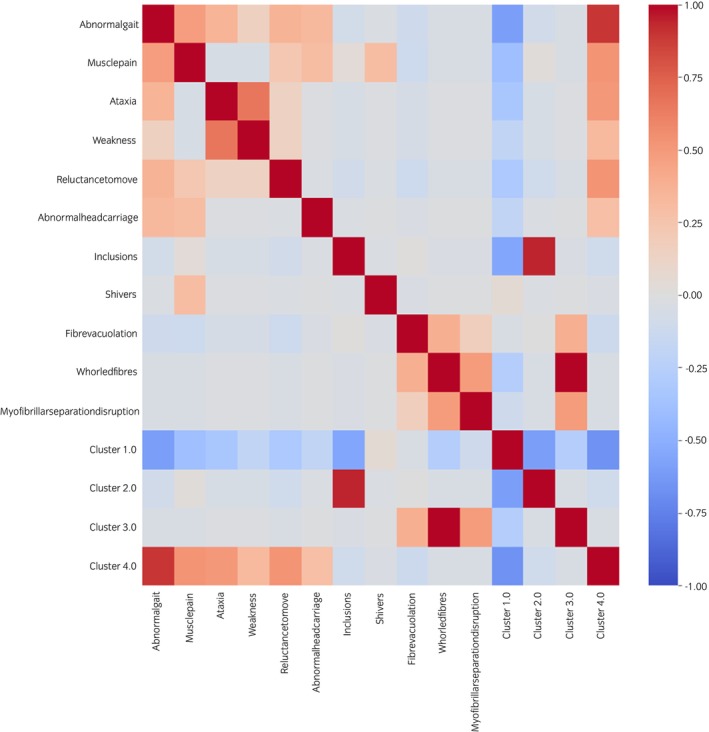
Heatmap of Spearman's correlations between the shortlisted significant and suggestive variables, and specific *k*‐means assigned phenotypic subtype. Stronger positive correlations are increasingly red with stronger negative correlations increasingly blue. Cluster 1 represented the classic RER phenotype, and Clusters 2–4 the non‐classic EAMS group.

Two feature selection methods were used, resulting in a longlist of 34 variables across groups. 64% of variables from *SelectKBes*t were also identified using *ExtraTreesClassifier*. Five variables were significant using *χ* ^2^ testing at a *p* < 0.05 Bonferroni corrected threshold (*p* < 0.0015), and a further five were suggestive using a nominal *p* < 0.05 (Table S3). Phenotypic subtype 1 (*n* = 87) was not associated with any variables, but each of the other three phenotypic subtypes did have associated variables. Phenotypic subtype 2 (*n* = 9) was associated with myofibre inclusions and the neurological sign colloquially referred to as ‘shivers’^51^, ^52^; phenotypic subtype 3 (*n* = 2) was associated with the histological signs of whorled fibres and myofibrillar separation and/or disruption; and phenotypic subtype 4 (*n* = 11) was associated with gait abnormalities, weakness, ataxia or ataxia‐like signs, reluctance to go forward and muscle pain. The Spearman's correlations between the significant variables and the four phenotypic subtypes are shown in Figure 3.

As the lack of specific associated variables in phenotypic subtype 1 aligned with previous descriptions of RER as being non‐specific in both clinical and histological signs beyond simply repeated episodes of ER, this phenotypic subtype was termed the ‘classic RER’ subtype. The other three phenotypic subtypes were then grouped as a combined ‘non‐classic’ group, termed exercise‐associated myopathy syndrome (EAMS), and comparisons were repeated between the classic RER and combined non‐classic EAMS subtypes (Table S3). While some histological features were significantly different between the four phenotypic subtypes, when the classic RER subtype was compared against the non‐classic EAMS subtypes (consisting of phenotypic subtypes 2, 3 and 4), only clinical signs remained significant or suggestive.

Histological and clinical history variables were then compared with the loadings from the PCA, which are illustrated in biplots in Figure S3. Muscle pain, abnormal gait, stiffness, weakness and ataxia are highly influential on the first three PCs, with at least one of these variables in each PC with loadings of greater than 0.2 or less than −0.2. Therefore, these variables account for large proportions of variation in the dataset.

No significant differences in serum CK activity scores were found (*p* = 0.926 and *p* = 0.638, respectively; Figure S2), either between all four phenotypic subtypes (Kruskal–Wallis test) and then between classic RER and non‐classic EAMS subtypes (two‐sided Mann–Whitney *U* test) in 49 horses that had suitable measurements.

#### 3.1.2 Validation using reduced input variables

While only 109 horses with complete signalment, clinical history and histological data were available, there were 196 horses with clinical history data available. Therefore, to validate the phenotype clustering results in the initial, well‐phenotyped 109 horses, the same analyses were re‐run using two validation sets based on clinical data only (Set V1, with the shortlisted clinical variables identified above, and Set V2, with all clinical variables) of these 196 horses.

In Set V1, PC1, PC2 and PC3 accounted for 16.1%, 13.7% and 13.1% of the variation in the dataset, while in Set V2, the first three PCs accounted for 5.98%, 5.91% and 5.48% of the variation, respectively. As in the Set 1 analysis, neither optimal *k* elbow plot had a decisive elbow (Figure S4). For Set V1, we therefore used the same number of phenotypic subtypes as in the Set 1 analysis (4), but in Set V2, there was a slight flattening of the trendline between 5 and 6, so 5 was used as the value for *k*. As in the Set 1 analysis, in both Set V1 and Set V2, a phenotypic subtype that had no associated variables except for ER episodes was present: in both Set V1 and Set V2 this was phenotype cluster 2, with the non‐classic EAMS subtype consisting of the combined other phenotypic subtypes in each Set (biplots are shown in Figure S5).

In Set V2, variables were longlisted as for the Set 1 analysis, this time selecting the top 15 scores from each method and creating a shortlist of 11 significant variables (Table S4). This list partially overlapped with the Set 1 associated variables, with abnormal gait, muscle pain, ataxia and shivers appearing on both lists. Presence of abnormal gait, muscle pain, ataxia, reluctance to go forward and weakness were all significantly different both between four phenotypic subtypes and between classic RER versus non‐classic EAMS subtypes in the Set V1 analysis (Table S4), with whorled fibres the only significant histological feature.

The Spearman's correlations of significant variables identified from Chi‐squared tests and the phenotypic subtypes identified from *k*‐means clustering in both sets are presented in Figure S6. Muscle pain, abnormal gait and ataxia were also significant in these validation analyses, as were some related clinical signs in Set V2 such as poor performance and lethargy. To compare correlations across the Set 1 analysis and the two validation sets, a heatmap of Spearman's correlations of the significant clinical signs across analyses were compared against the classic RER versus non‐classic EAMS subtype from each analysis (Figure S7). Broadly, the classic RER and non‐classic EAMS subtypes correlated between analyses, with similar patterns in associated clinical signs.

Serum CK scores were then compared between all four phenotypic subtypes (Kruskal–Wallis test) and then between classic RER and non‐classic EAMS subtypes (two‐sided Mann–Whitney *U* test) in 98 and 99 horses in respective sets. These comparisons were not significantly different in Set V1 (*p* = 0.513 and *p* = 0.094, respectively), but were when comparing between classic RER and non‐classic EAMS subtypes in Set V2 (*p* = 0.221 and *p* = 0.017, respectively), with the classic RER horses having a higher CK (Figure S8).

To directly compare horses that were assigned to the classic RER or non‐classic EAMS subtypes in the Set 1 analysis versus the two validation sets, contingency tables were produced (Table 2). Each validation set broadly agreed with the initial analysis but Set V1 had a higher percentage agreement at 88.1%–76.1%. Receiver operating characteristic (ROC) curves were also produced, treating the Set 1 analysis as the ‘true’ values. Area under the curve (AUC) was also slightly higher in Set V1 than Set V2 (0.79–0.78).

**TABLE 2:** Agreement between Set 1 and Set 2 classic RER versus non‐classic EAMS subtypes and those of the initial analysis.

		Set V1		Set V2	
		EAMS	RER	EAMS	RER
Set 1	EAMS	14	8	18	4
	RER	5	82	22	65
Percentage agreement with classic RER subtype		88.1%		76.1%	
AUC from ROC curve between Set 1 and validation set		0.79		0.78	

*Note*: The classic RER subtype was the same as phenotypic subtype 1 in the Set 1 analysis and represented phenotypic subtype 2 in both Set V1 and Set V2. The non‐classic EAMS subtype in the Set 1 analysis consisted of phenotypic subtypes 2, 3 and 4, while in Set V1 it consisted of phenotypic subtypes 1, 3 and 4, and in Set V2 it consisted of phenotypic subtypes 1, 3, 4 and 5.

Abbreviations: AUC, area under the curve; ROC, receiver operating characteristic.

#### 3.1.3 Validation using hierarchical clustering

The hierarchical clustering of Set 1 horses using all clinical and histopathological data did not distinguish well between classic RER and non‐classic EAMS subtype horses, although the first branch of the dendrogram contained notably more EAMS horses than the latter branches. However, when combined histopathological and clinical history variables were analysed, a cluster formed consisting of muscle pain, abnormal gait, reluctance to go forward and lethargy, which were identified as shortlisted variables in the *k*‐means clustering analyses (Figure S9).

Both Set V1 and Set V2 were better distinguished between classic RER and non‐classic EAMS subtypes than in the initial set using all clinical and histological data, and broad agreement was seen between *k*‐means and hierarchical clustering (Figure S10). Similar clustering of muscle pain, abnormal gait and reluctance to go forward was noted, indicating that these clinical signs repeatedly group together across multiple datasets and clustering methods.

### 3.2 Histological comparison of phenotype sub‐groups

Of the 69 muscle biopsy samples assessed in this study, no samples had patterns of desmin accumulation that appeared as described by Valberg et al.^36^, ^37^ This included all of the ‘positive control’ samples that were selected based on myofibrillar pathological features, and the non‐classic EAMS subtype horses with a similar clinical phenotype to that described as equine MFM. This, supported by the finding that none of the 1096 cases submitted to the CNMDL biopsy service were given a diagnosis of MFM, indicates that the disease described by Valberg et al.^36^, ^37^ is rare, or non‐existent in UK/European horses, and distinct from the therefore novel disease phenotype described here.

In some samples, a diffuse residue from the desmin IHC was seen, rather than the protein aggregation as described in MFM. This had an appearance similar to artefactual staining which is common with DAB IHC. Table S5 illustrates Spearman's correlations between apparent desmin aggregate severity score and various key histopathology and scores for common causes of artefact in frozen muscle sections. None of these correlations was strong: the strongest being negative associations between desmin aggregate score and both freeze artefact (Spearman's rho = −0.320) and fibre necrosis scores (Spearman's rho = −0.317) (*p* = 0.017 and *p* = 0.008, respectively). Figure S11 illustrates Spearman's correlations between the various desmin variables and a range of other variables, including breed and disease type dummy variables. Only three variables showed moderate to high correlations with desmin variables—the ‘occasional accumulation of desmin’ pattern correlated both with freeze and with saline artefact, while the ‘central accumulation of desmin’ pattern correlated very highly with the ‘rod body’ score. However, both latter correlating variables only appeared in one horse, which happened to be the same animal—and is thus highly unlikely to be representative of any kind of a more widely applicable, causal association.

Variables were filtered for inclusion in the multinomial and logistic regression models, and backward feature elimination applied, with no variance inflation factor above 5 in any step. The final models can be seen in Table 3. None of the logistic regression models between classic RER and non‐classic EAMS groups had good predictive ability (as evaluated by the pseudo‐*r* ^2^ and model *p*‐values), so the significant variables in these final models are not well supported, while in contrast, the multinomial models had low pseudo‐*r* ^2^ (0.152–0.205) but were significant (*p* = 0.003–0.032). Across all breeds, there were no variables significantly different between the classic RER and non‐classic EAMS groups, however when Thoroughbreds were excluded, fibre hypertrophy and peripheral accumulation of mitochondria became significant and breed became suggestive in this model. The significant variables are presented as bar plots in Figure S12.

**TABLE 3:** Model metrics and significant histological variables in regression models with disease group or myopathy subtype as the outcome variable.

Model	Breed	Pseudo *r* ^2^	Model *p* value	Final variables	Variable coefficient ± SE (*p*‐value)		
					Negative control	Non‐classic EAMS	Myofibrillar abnormalities
Multinomial	Included	0.182	**0.01**	TB	0.277 ± 0.625 (0.658)	0.240 ± 0.755 (0.751)	−0.070 ± 0.988 (0.943)
				Arabian	−0.770 ± 1.207 (0.524)	−1.056 ± 1.202 (0.380)	1.271 ± 0.980 (0.195)
				Diffuse lobulated fibres	1.376 ± 0.681 **(0.043)**	−1.458 ± 0.903 (0.107)	0.327 ± 1.003 (0.744)
				Myofibrillar aggregate score	2.205 ± 1.095 **(0.044)**	0.315 ± 1.323 (0.812)	2.102 ± 1.167 (0.072)
				Polyglucosan score	−2.202 ± 1.107 **(0.047)**	−1.008 ± 1.048 (0.336)	−0.829 ± 1.132 (0.464)
				Endomyseal lipid score	−0.704 ± 0.440 (0.109)	−0.119 ± 0.440 (0.755)	−1.788 ± 0.687 **(0.009)**
Multinomial	Not included	0.151	**0.003**	Diffuse lobulated fibres	1.506 ± 0.628 **(0.017)**	−1.273 ± 0.866 (0.142)	0.265 ± 0.898 (0.767)
				Myofibrillar aggregate score	2.251 ± 1.063 **(0.034)**	0.430 ± 1.289 (0.739)	1.893 ± 1.103 (0.086)
				Polyglucosan score	−2.300 ± 1.101 **(0.037)**	−0.990 ± 1.042 (0.342)	−0.762 ± 1.020 (0.455)
				Endomyseal lipid score	−0.725 ± 0.408 (0.075)	−0.218 ± 0.341 (0.523)	−1.483 ± 0.608 **(0.015)**
Logistic	Included	No final significant variables in the model					
Logistic	Not included	Infinite	Not estimable	Glycogen score	0.270 ± 0.137 **(0.049)**		
Multinomial (WB & Arabian)	Included	0.205	**0.03**	Arabian	−2.610 ± 1.391 (0.061)	−2.719 ± 1.429 (0.057)	−1.418 ± 1.117 (0.204)
				Fibre hypertrophy score	1.397 **±** 0.644 **(0.030)**	1.373 ± 0.645 **(0.033)**	1.226 ± 0.639 (0.055)
				Myofibrillar aggregate score	3.167 ± 1.530 **(0.038)**	1.111 ± 1.314 (0.098)	1.403 ± 1.047 (0.18)
				Polyglucosan score	−4.758 ± 2.305 **(0.039)**	−2.004 ± 1.266 (0.113)	−1.503 ± 1.000 (0.133)
				Peripheral mitochondrial accumulation score	−1.386 ± 0.649 **(0.033)**	−1.410 ± 0.656 **(0.031)**	−2.109 ± 0.976 **(0.031)**
Logistic (WB & Arabian)	Included	Infinite	>0.9	Arabian	−0.033 ± 1.010 (0.974)		
				Internalised nuclei score	0.401 ± 0.204 (**0.049)**		
Logistic (WB & Arabian)	Not included	Infinite	Not estimable	Internalised nuclei score	0.391 ± 0.183 **(0.032)**		

*Note*: *p* values in bold represent variables significant at an alpha of 0.05; *p* values in italics represent suggestive variables at a threshold of 0.1.

Abbreviation: SE, standard error.

The loading plots from the PCA are presented in Figure S13. Across the three PCs, groupings of variables are noted. Freeze artefact, saline artefact and occasional accumulation of desmin consistently group together, reflecting the correlation between these variables identified in Figure S13; whole‐fibre variables such as generalised atrophy, fibre atrophy, fibre angular atrophy and fibre size variation also consistently grouped. Moreover, in some plots, fibre necrosis and peripheral accumulation of mitochondria, and subcellular variables such as polyglucosan, myofibrillar aggregates, myofibrillar loss and/or separation, and rimmed vacuoles, in some plots with rod bodies, also consistently grouped together. The datapoints themselves did not cluster by disease group.

## 4 DISCUSSION

Increasingly, clinically heterogeneous complex diseases are being approached as syndromes with multiple disease subtypes that have their own genetic risk factors. In humans, studies have shown distinct phenotypic subtypes in type 2 diabetes,^53^, ^54^, ^55^, ^56^, ^57^ polycystic ovary syndrome,^58^ preterm birth syndrome,^59^ acute respiratory distress syndrome,^60^, ^61^, ^62^ and heart failure^63^ among other complex diseases. This substantive and growing body of work indicates that this subtype clustering approach is essential for studying complex diseases. Consequently, the overall aim of this work was to determine whether distinct subtypes of exercise‐associated myopathies can be defined based on the clinical history and histological data, using submissions from a referral neuromuscular diagnostic biobank, and methods that made no prior assumption of clinically important variables. Our algorithms identified a recurring phenotype, distinct from the classical RER phenotype, consisting of the clinical signs of ataxia, weakness, muscle pain, abnormal gait or reluctance to go forward under saddle that was present across clustering methods and datasets—a phenotype we have chosen to term ‘exercise‐associated myopathy syndrome’ (EAMS), to distinguish this subtype from RER.

Characterising subgroups within complex disease syndromes has been uncommonly used in animals. In horses, a research group described use of PCA quantitatively to score equine metabolic syndrome (EMS) phenotypes in Arabian horses^64^: the first two factor scores could predict accurately between healthy horses, high EMS risk and high pars pituitary intermedia dysfunction (PPID) risk. The particular benefit of this method was the small number of easily obtainable quantitative measures required for prediction, making this an appealing method of risk assessment in the clinic. Similarly, our study has also demonstrated a small number of clinical signs that allow exercise‐associated myopathy subtypes to be simply delineated, although with the aim of classification rather than creation of a quantitative phenotype. This presents an interesting avenue for potential future work, perhaps including further use of quantitative clinically‐relevant variables such as serum CK activity or other biomarkers.

In this study, we identified a consistent classic RER subtype with no significant associations with any histological or clinical history variables. Our algorithms identified at least three non‐classic phenotypic subtypes in the different datasets, however the sample size within these different phenotypic subtypes was too small to confidently describe these as distinct disease subtypes; we therefore also compared the classic (RER) and combined non‐classic (EAMS) subtypes. However, across multiple clustering methods (PCA, *k*‐means and hierarchical clustering) and different horse groupings (the initial set was almost doubled in size in the validation sets), similar clinical signs repeatedly described EAMS.

Identification of similar described clinical signs across multiple methods with different mathematical basis and different groups of horses indicates that the signs of ataxia, weakness, muscle pain, abnormal gait and reluctance to go forward under saddle contribute to a distinct EAMS phenotype. Weakness is a common, non‐specific feature of many human myopathies including the various forms of MFM,^65^ and is also reported in horses with other genetic neuromuscular diseases such as hyperkalaemic periodic paralysis (HYPP).^66^, ^67^ It is not however a phenotypic feature of ER subtypes in humans (such as those with ER‐associated *RYR1* mutations^68^): instead in human ER, muscle hypertrophy and improved athletic ability are common. In contrast, weakness was reported associated with EAMS in horses in this study and further, some horses were regarded as ataxic by the referring veterinary surgeon. While perceived ataxia in these horses could reflect true proprioceptive dysfunction, it might also have reflected instead paresis as the two signs can be difficult to define and distinguish, especially when signs are mild.^69^ Lameness has been found in 34.1% of Standardbreds with RER previously,^13^ and while this could be partially attributed to clinical muscle pain from ER episodes, the clustering of gait abnormalities as part of a distinct phenotype indicates that there is likely something further underlying this clinical sign. Reluctance to go forward under saddle could be due to post‐episode pain or subclinical muscle pain, but in most clinical histories it was implied to be a chronic problem, which would seem at odds with previous evidence of a conferred performance advantage in Standardbred horses with RER.^13^ Taken together, it would seem that these clinical features describe EAMS phenotype(s) distinct from classic RER with likely differing underlying pathophysiology and signs. We also consider it possible (perhaps probable) that this additional myopathic syndrome or syndromes, though most readily identified by owners, trainers or their attending clinician during or associated with exercise, actually represents chronic (perhaps often subclinical) disease that is also present at rest but is exacerbated by exercise; this is supported by the accompanying clinical signs involving gait, strength and/or movement.

Similar, linked clinical features have previously been reported as a possible myopathy subtype,^36^, ^37^ in particular, in association with a disorder termed MFM. However, our blinded histological evaluation of classic and non‐classic groups identified no horses with the histopathological feature of desmin aggregation, which is the defining feature of this reported disorder. As such, this non‐classic EAMS subtype identified in our current study seems therefore to be novel. However, equine MFM has previously been diagnosed following identification of as few as five or six myofibres containing desmin aggregates within an entire muscle biopsy transverse section.^36^, ^70^ Assuming a muscle fibre diameter of 50 μm, and a biopsy section of 5 × 5 mm, this equates to a proportion of desmin aggregated fibres in a single section of approximately 0.05% (1 in 2000). It is possible therefore that, if a horse in this study had as few affected fibres proportionally as previously described, the assessed muscle biopsy samples simply did not capture myofibres containing these aggregates or they were missed. However, the biological significance of so few affected fibres in a standard biopsy section is questionable: more than 75% of fibres are affected in many humans with MFM,^38^, ^39^ and low levels of desmin and other cytoskeletal protein aggregation are often seen as non‐specific features of non‐desmin‐related myopathies such as inclusion body myositis,^71^ central core^72^ and minicore myopathies.^73^ Indeed, desmin accumulation is a typical feature of muscle regeneration^74^ which would be a non‐specific feature of most myopathies characterised by muscle damage and elevated serum CK activity. It seems unlikely then that horses with so few affected fibres have any specific desminopathy, or that so few affected fibres have sufficient cumulative effect to produce the severity of clinical signs described by the referring veterinary surgeons. Regardless, due to the lack of pathological desmin staining in these tested samples and considering the lack of any previously diagnosed MFM cases in this UK database, it would appear that the EAMS subtype identified in our study is not equine MFM, but instead, a distinct disease phenotype.

The only histological variables that were significantly different between classic RER and non‐classic EAMS were fibre hypertrophy and peripheral mitochondrial accumulation, both of which were only significant when Thoroughbreds were excluded. It is possible, considering that breed was approaching significance between these groups, the former effect is caused by differences in exercise and training (which were not known for these samples) and the latter is linked to breed rather than disease processes. Muscle hypertrophy, or the addition of myofilaments in the myofibre, can be directly caused by training as well as by compensatory load on myofibres during disease,^75^, ^76^ and data on exercise levels in samples from the biobank were unavailable. However, as neither fibre atrophy nor other non‐specific myopathic signs were significantly different between groups, the difference in muscle fibre hypertrophy seen here is unlikely due to compensatory load. Arabian horses have a greater proportion of oxidative fibres than breeds such as Warmbloods and Thoroughbreds,^77^ and unlike in humans where peripheral, subsarcolemmal mitochondrial accumulation—so called ‘ragged red’ fibres—are indicative of a mitochondrial myopathy, peripheral accumulation of mitochondria can be considered normal in horses.^78^ While a significant difference between RER and EAMS subtypes could indicate a fibre type shift due to necrosis of specific fibre types and possible subsequent disease adaptations, or even some role of the mitochondria in the disease processes of these disease subtypes, this effect seems more likely simply to be attributed to fibre type differences between breeds.

In our study, none of the histological stains, nor their combination, from a typical neuromuscular diagnostic panel identified differences in histological phenotype between classic RER and non‐classic EAMS disease groups. It is possible that further study using selected non‐standard immunohistochemistry stains might identify differences between these groups; currently however, both subtypes have a non‐specific myopathic histological appearance. This is the case in many human myopathies, including human MFM, where desminopathies, filaminopathies, ZASPopathies, αβ‐crystallinopathies, myotilinopathies and other MFMs have a heterogeneous and poorly‐delineated histological phenotype,^39^, ^41^, ^65^ and in human ER, where a large range of mutations across more than 30 genes cause ER.^68^, ^79^ Further genetic study of these distinct phenotypes might help sub‐categorise idiopathic equine exercise‐associated myopathies facilitating more directed histopathological comparisons.

Future prospective validation of both the non‐classic EAMS subtype and the small sample size phenotypic subtypes identified in a larger, biologically independent, validation cohort would be of benefit in the next stage in characterising EAMS subtypes. As the RVC CNMDL referral neuromuscular diagnostic biobank is likely one of the largest in Europe, sourcing sufficient phenotyped European samples with a similar demographic might prove challenging. However, clinical history data should prove easier to obtain: indeed, this study suggests that clinical history is more useful for delineating phenotypic subtypes than histopathological criteria. However, independent, preferably blinded, examination by a single or small team of skilled clinicians, adept at distinguishing and accurately defining neurological and musculoskeletal gaits appears important as does future use of a standardised questionnaire for full case characterisation. However, confirmation that a horse has non‐specific histopathological features that confirm the myopathy, would delineate affected horses from those with shared, vague and often poorly defined clinical signs (such as weakness) that might otherwise reflect unrelated involvement of other body systems. As such, muscle biopsy confirmation remains a key step in disease characterisation.

In summary, using clinical history and histopathological features from biobanked samples, we have identified a distinct exercise‐associated myopathy phenotypic subtype we have termed EAMS, consisting of horses with weakness, perceived ataxia, muscle pain, gait abnormalities and reluctance to go forward under saddle. This was validated using different horse groupings, variable groupings, and clustering methods, with similar clinical signs consistently identified. This EAMS subtype is distinct from the disorder referred to as equine MFM; indeed, MFM was not detected in these European horses. Further, there were no histopathological features that distinguished between classic RER and non‐classic EAMS across breeds, indicating that this novel subtype has a similar non‐specific myopathic appearance using a standard muscle staining panel. While it remains unclear whether this novel subtype represents a single disease entity, and whether the practitioner‐described clinical signs represent true associations, co‐variates or simply, improper classifications, its recognition for both future genetic and clinical studies might improve study power and help end the long delay in research progress with exertional myopathic syndromes of horses.

## FUNDING INFORMATION

This work was funded by the Royal Veterinary College's Mellon Fund for Equine Research and by the RVC Comparative Neuromuscular Diseases Laboratory diagnostic biopsy service.

## CONFLICT OF INTEREST STATEMENT

The authors declare no conflicts of interest.

## AUTHOR CONTRIBUTIONS

**Victoria Lindsay‐McGee:** Conceptualization; data curation; formal analysis; investigation; methodology; software; validation; visualization; writing – original draft; writing – review and editing. **Claire Massey:** Investigation; methodology; resources; writing – review and editing. **Ying Ting Li:** Investigation; methodology; resources; writing – review and editing. **Emily L. Clark:** Conceptualization; funding acquisition; project administration; supervision; writing – review and editing. **Androniki Psifidi:** Conceptualization; funding acquisition; methodology; project administration; resources; supervision; validation; writing – review and editing. **Richard J. Piercy:** Conceptualization; formal analysis; funding acquisition; investigation; methodology; project administration; resources; supervision; validation; writing – original draft; writing – review and editing.

## DATA INTEGRITY STATEMENT

V. Lindsay‐McGee had full access to all the data in the study and takes responsibility for the integrity of the data and the accuracy of the data analysis.

## ETHICAL ANIMAL RESEARCH

All work was conducted with the approval of the Royal Veterinary College's Clinical Research Ethical Review Board (CRERB, reference 2018 1834‐2) and Social Science Research Ethical Review Board (SSRERB, reference SR2018‐1799).

## INFORMED CONSENT

Explicit owner consent for animals' inclusion in the study was not stated.

### PEER REVIEW

The peer review history for this article is available at https://www.webofscience.com/api/gateway/wos/peer-review/10.1111/evj.14128.

## Supporting information


**Figure S1.** Elbow plots.

**Figure S2.** Violin plots showing differences in distribution of binned serum CK activity scores in 49 horses in Set 1.

**Figure S3.** Biplot of principal components from the PCA of Set 1 (*n* = 109).

**Figure S4.** Elbow plots of: variance explained by principal component.

**Figure S5.** PCA biplots of the first three principal components.

**Figure S6.** Heatmap of pairwise Spearman's correlations.

**Figure S7.** Heatmap of pairwise Spearman's correlations between clinical variables significant between classic RER and combined non‐classic EAMS subtypes from the Set 1 analysis.

**Figure S8.** Violin plots showing difference in: distribution of binned serum CK activity scores between *k*‐means assigned clusters (phenotypic subtype).

**Figure S9.** Vertical: hierarchical clustering using Ward's method of 109 Set 1 RER horses based on histological and clinical variables. Horizontal: hierarchical clustering using Ward's method of 45 clinical and histological variables based on 109 RER horses.

**Figure S10.** (A) Hierarchical clustering in Set 1; (B) Hierarchical clustering in Set 2.

**Figure S11.** Heatmap of Spearman's correlations of desmin‐related variables with selected signalment and histological variables from the histological comparison.

**Figure S12.** Bar plot of significant variables in the final models.

**Figure S13.** Biplots of PCA scores and the loadings of variables with loadings >0.2 or <−0.2 on either of the plotted principal components.

**Table S1.** Signalment, clinical history, biochemistry, environment and histological variables recorded for analysis of the Comparative Neuromuscular Diseases Laboratory diagnostic service records.

**Table S2.** Variables scored for blinded histology study.

**Table S3.** Significance of longlist variables between *k*‐means phenotypic subtypes, and between classic RER and non‐classic EAMS subtype.

**Table S4.** Significance of longlist variables between *k*‐means phenotypic subtypes, and between classic RER and non‐classic EAMS subtypes, in Set 1 and Set 2.

**Table S5.** Correlation between histological variable scores and desmin aggregate score.

## Data Availability

The data that support the findings of this study are available from the corresponding author upon reasonable request: Open sharing exemption granted by editor for this retrospective clinical report.
