# Supplementary material for: Characterisation of phenotypic patterns in equine exercise‐associated myopathies
Source: Equine Vet J. 2024 Jul 5;57(2):347–61. doi: 10.1111/evj.14128 (PMC11807944; doi:10.1111/evj.14128)

Equine Veterinary Journal BEVA

**Figure S1:** A) Elbow plot of variance explained per principal component calculated from the PCA of Set 1; B) Elbow plot for optimal k in Set 1, using sum of squared distances by value for k in k-means clustering of 109 horses with exercise-related myopathies. Elbow plot of variance explained demonstrate how much additional variation in the dataset each additional PC explains, whilst elbow plots for optimal k identify how much of the variation in the dataset is explained using each number of clusters (k) in a k-means clustering analysis. In this analysis, 4 PCs did not add a lot more variance explained to 3 PCs, and 5 clusters did not explain considerably more variation than 4.

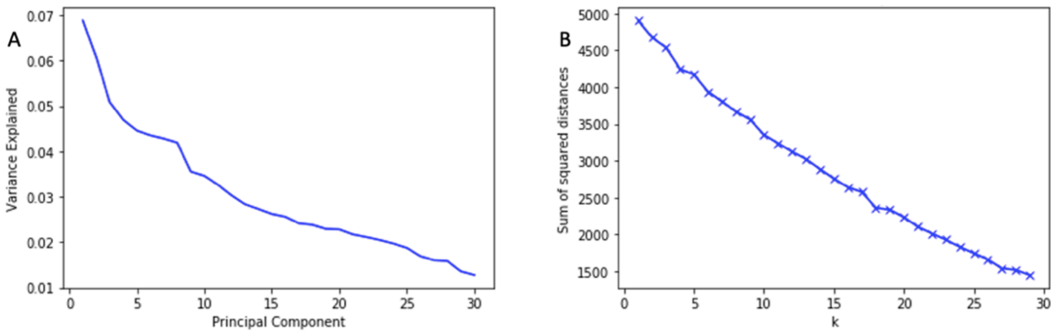

Supplement: Supplementary file 1 — Figure S1. Elbow plots. [file EVJ-57-347-s011.pdf]
