# Supplementary material for: Characterisation of phenotypic patterns in equine exercise‐associated myopathies
Source: Equine Vet J. 2024 Jul 5;57(2):347–61. doi: 10.1111/evj.14128 (PMC11807944; doi:10.1111/evj.14128)

Figure S2: Violin plots showing differences in distribution of binned serum CK activity scores in 49 horses in Set 1 by A) k-means assigned cluster (phenotypic subtype); and B) classic RER versus combined non-classic EAMS subtypes. Individual datapoints are given as grey circles. No significant differences in binned CK score were identified using either grouping.

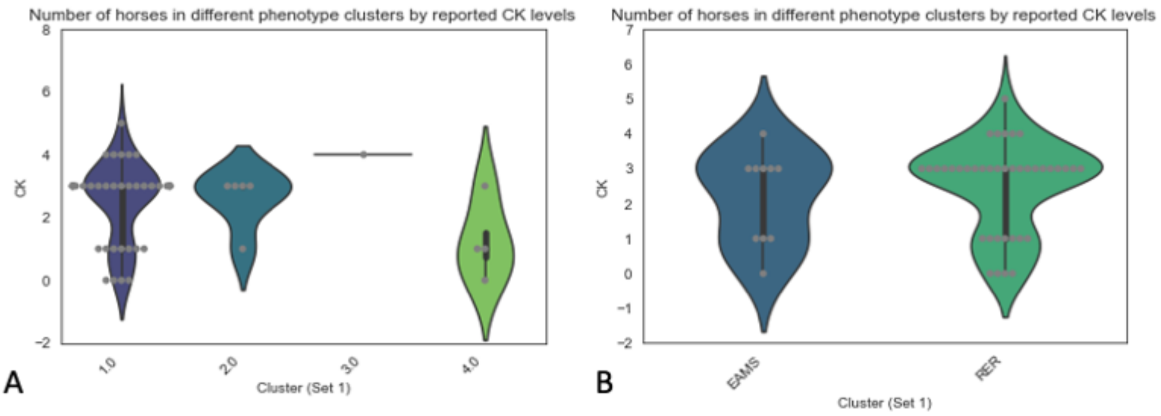

Supplement: Supplementary file 2 — Figure S2. Violin plots showing differences in distribution of binned serum CK activity scores in 49 horses in Set 1. [file EVJ-57-347-s014.pdf]
