# Supplementary material for: Characterisation of phenotypic patterns in equine exercise‐associated myopathies
Source: Equine Vet J. 2024 Jul 5;57(2):347–61. doi: 10.1111/evj.14128 (PMC11807944; doi:10.1111/evj.14128)
Supplement: Supplementary file 3 — Figure S3. Biplot of principal components from the PCA of Set 1 (n = 109). [file EVJ-57-347-s001.pdf]

Equine Veterinary Journal BEVA

Figure S3:

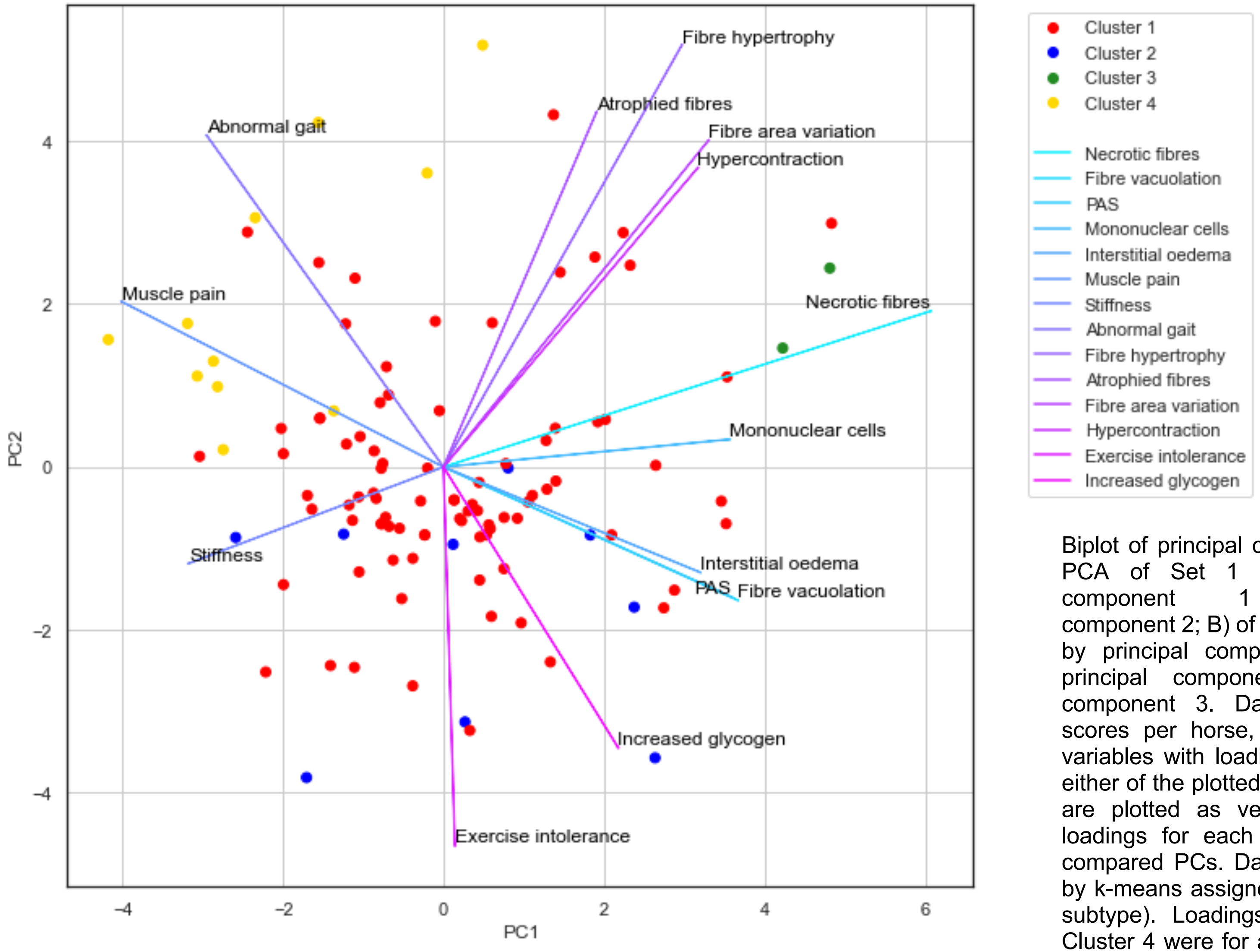

Biplot of principal components from the PCA of Set 1 (n=109). A) principal component 1 by principal component 2; B) of principal component 1 by principal component 3; and C) of principal component 2 by principal component 3. Datapoints show PCA scores per horse, and the loadings of variables with loadings >0.2 or <-0.2 on either of the plotted principal components are plotted as vectors relative to the two loadings for each variable on the two compared PCs. Datapoints are coloured by k-means assigned cluster (phenotypic subtype). Loadings in the direction of Cluster 4 were for abnormal gait, muscle pain, weakness, ataxia and stiffness whilst increased glycogen and exercise intolerance were loading towards Cluster 2.

B

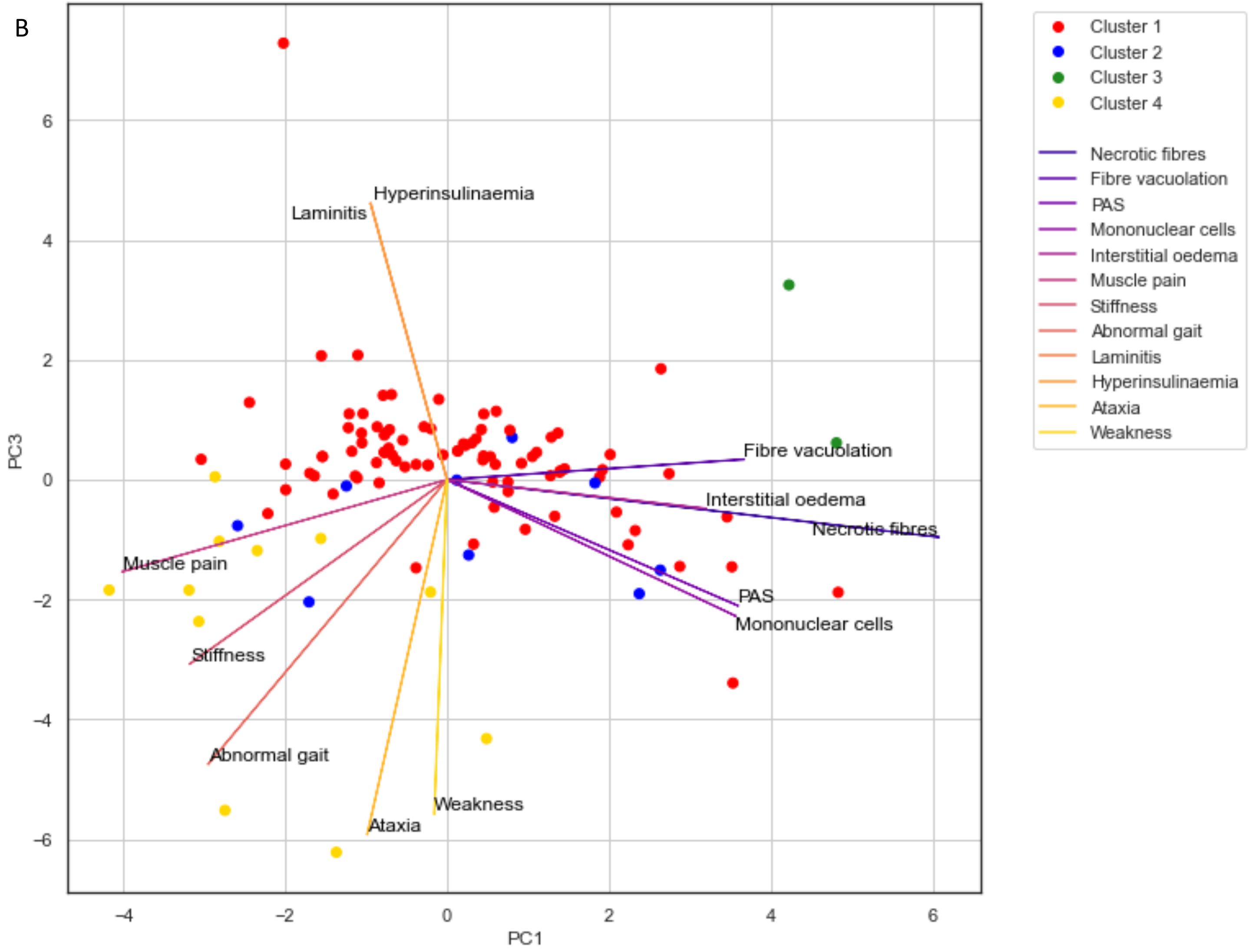
PC1
PC3
Hyperinsulinaemia
Laminitis
Fibre vacuolation
Interstitial oedema
Necrotic fibres
Muscle pain
PAS
Mononuclear cells
Stiffness
Abnormal gait
Weakness
Ataxia
Cluster 1
Cluster 2
Cluster 3
Cluster 4
Necrotic fibres
Fibre vacuolation
PAS
Mononuclear cells
Interstitial oedema
Muscle pain
Stiffness
Abnormal gait
Laminitis
Hyperinsulinaemia
Ataxia
Weakness

C

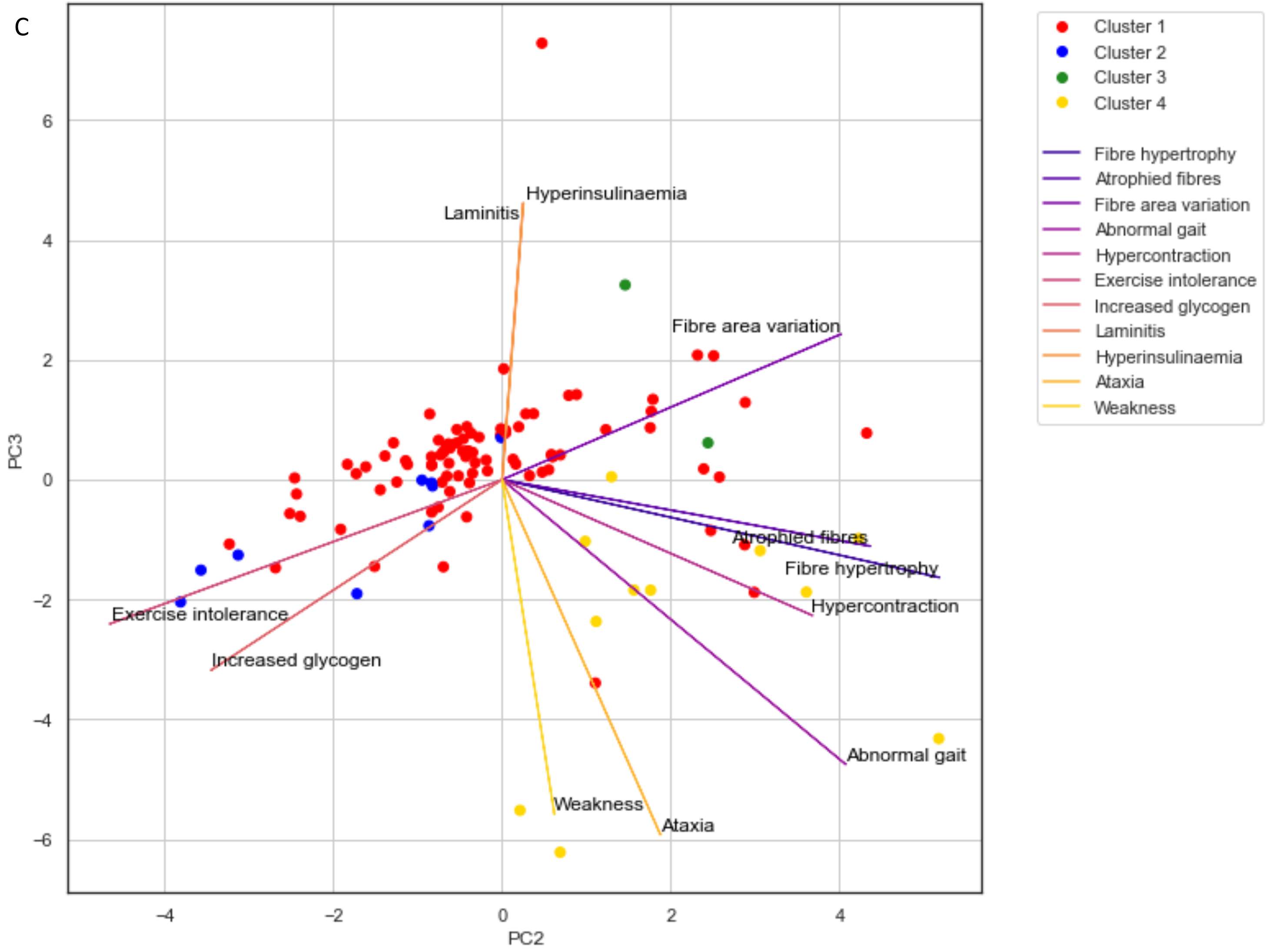

Hyperinsulinaemia
Laminitis
Fibre area variation
Atrophied fibres
Fibre hypertrophy
Hypercontraction
Exercise intolerance
Increased glycogen
Abnormal gait
Weakness
Ataxia
PC3
PC2
6
4
2
0
-2
-4
-6
-4
-2
0
2
4
Cluster 1
Cluster 2
Cluster 3
Cluster 4
Fibre hypertrophy
Atrophied fibres
Fibre area variation
Abnormal gait
Hypercontraction
Exercise intolerance
Increased glycogen
Laminitis
Hyperinsulinaemia
Ataxia
Weakness
