# Supplementary material for: Characterisation of phenotypic patterns in equine exercise‐associated myopathies
Source: Equine Vet J. 2024 Jul 5;57(2):347–61. doi: 10.1111/evj.14128 (PMC11807944; doi:10.1111/evj.14128)

Equine Veterinary Journal BEVA

Figure S4: Elbow plots of: variance explained by principal component from the PCA for A) Set V1, using the shortlisted variables from the Set 1 analysis (n=196) and B) Set V2, using all clinical history variables (n=196); optimal k for k-means clustering, with sum of squared distances by value for k in C) Set V1, using the shortlisted variables from the Set 1 analysis (n=196) and D) Set V2, using all clinical history variables (n=196). Elbow plot of variance explained demonstrate how much additional variation in the dataset each additional PC explains, whilst elbow plots for optimal k identify how much of the variation in the dataset is explained using each number of clusters (k) in a k-means clustering analysis. In this analysis, there was no clear indicator of optimal k in Set V1, but 5 appeared to be optimal k for Set V2.

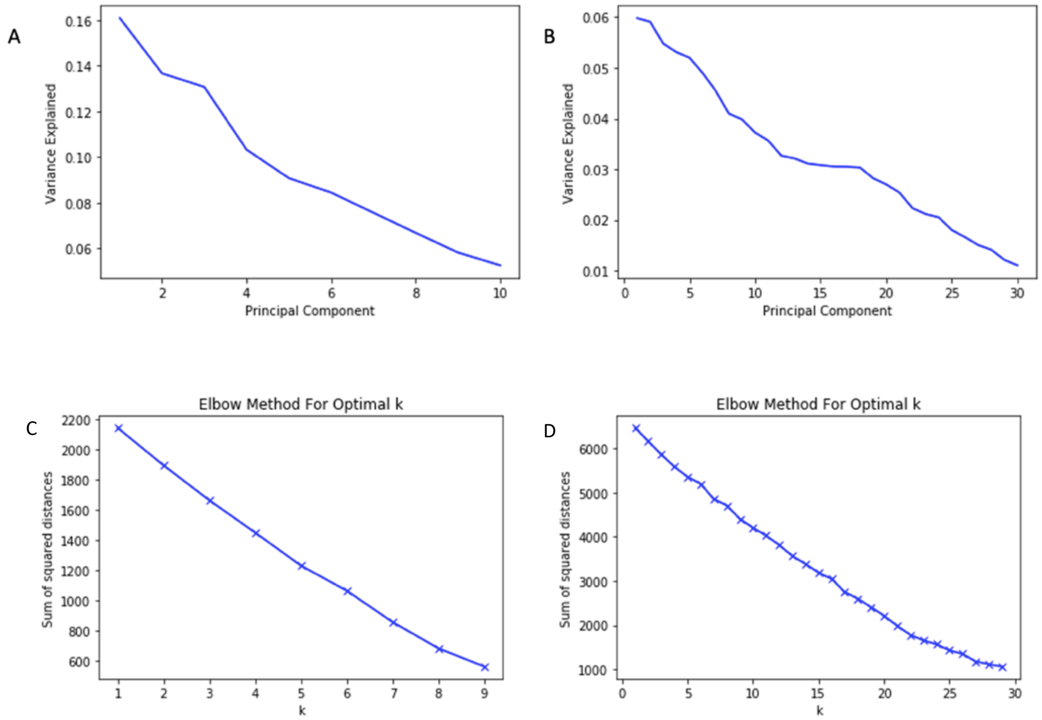

Supplement: Supplementary file 4 — Figure S4. Elbow plots of: variance explained by principal component. [file EVJ-57-347-s015.pdf]
