# Supplementary material for: Characterisation of phenotypic patterns in equine exercise‐associated myopathies
Source: Equine Vet J. 2024 Jul 5;57(2):347–61. doi: 10.1111/evj.14128 (PMC11807944; doi:10.1111/evj.14128)
Supplement: Supplementary file 5 — Figure S5. PCA biplots of the first three principal components. [file EVJ-57-347-s009.pdf]

Equine Veterinary Journal BEVA

Figure S5: PCA biplots of the first three principal components, with Set V1 on the upper diagonal and Set V2 on the lower diagonal. On the diagonal: kernel density estimator (KDE) plots of the distribution of samples on each principal component. Plots are coloured by: A) K-means assigned cluster (phenotypic subtype) per Set; B) Classic RER versus combined non-classic EAMS subtypes. In both Sets the classic RER subtype was the same as cluster (phenotypic subtype) 2, whilst the Set V1 non-classic EAMS subtype consisted of phenotypic subtypes 1, 3 and 4, and in Set V2 this was phenotypic subtypes 1, 3, 4 and 5.

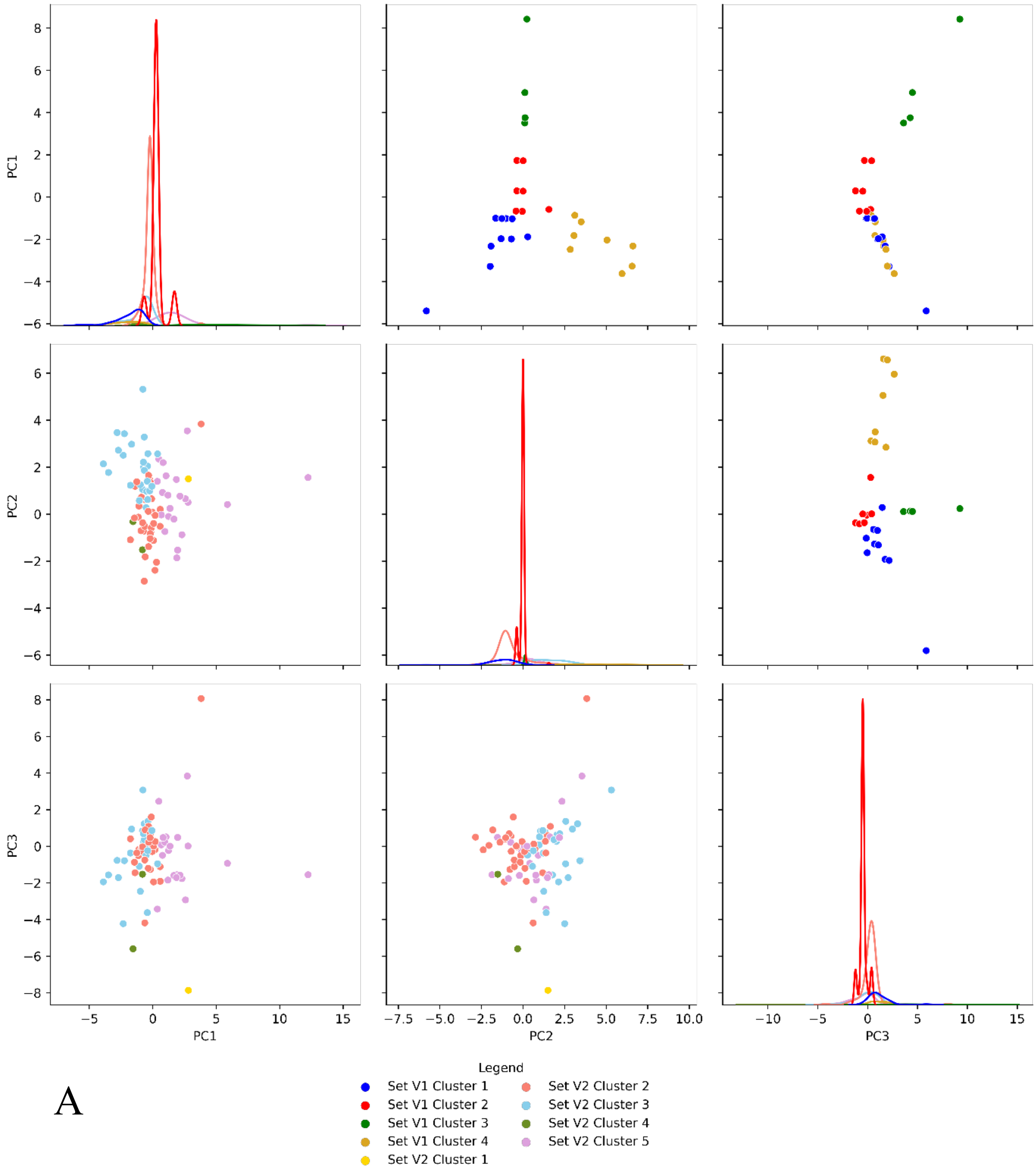

A

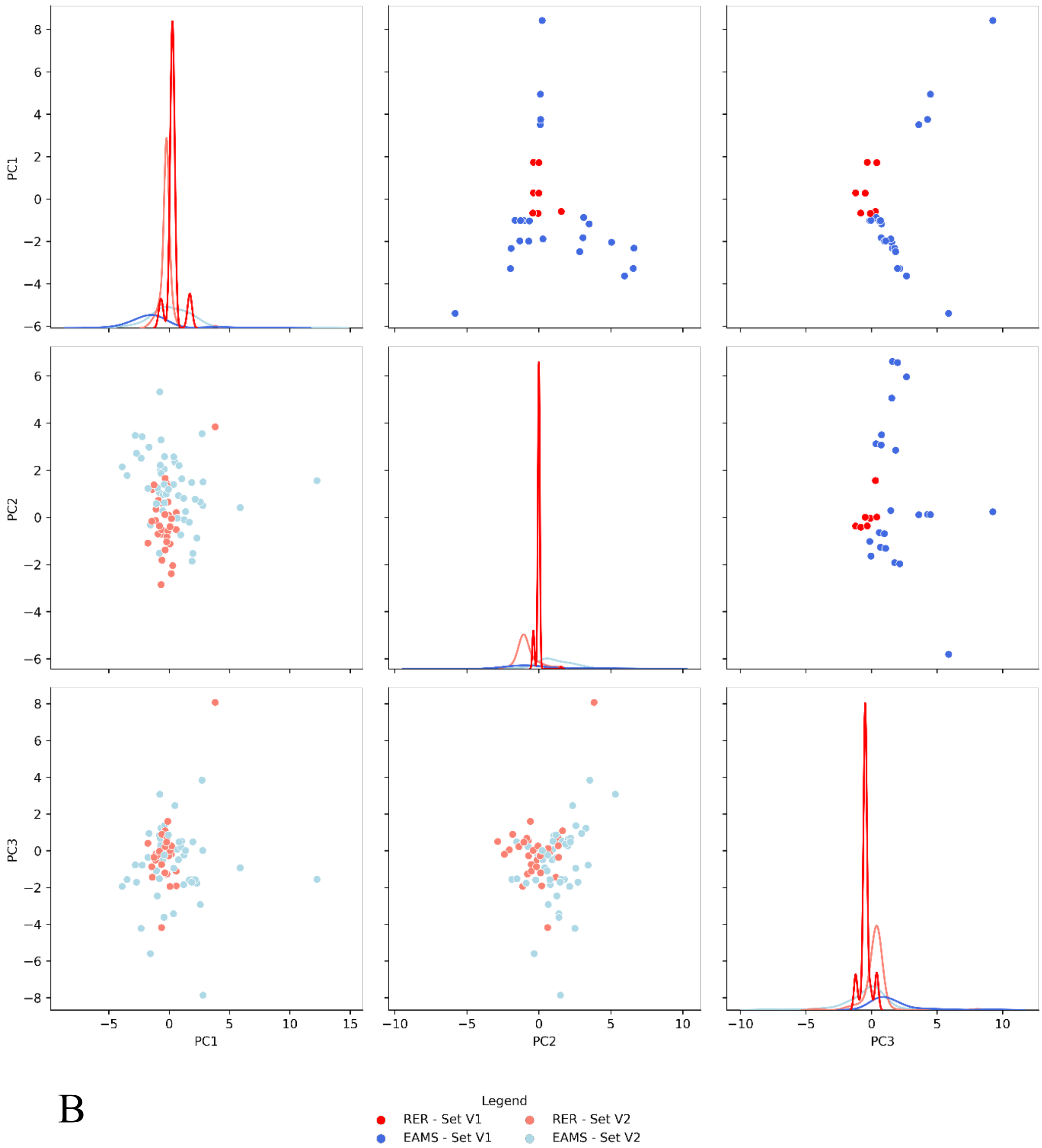

B
