# Supplementary material for: Characterisation of phenotypic patterns in equine exercise‐associated myopathies
Source: Equine Vet J. 2024 Jul 5;57(2):347–61. doi: 10.1111/evj.14128 (PMC11807944; doi:10.1111/evj.14128)
Supplement: Supplementary file 6 — Figure S6. Heatmap of pairwise Spearman's correlations. [file EVJ-57-347-s008.pdf]

Equine Veterinary Journal BEVA

Figure S6: Heatmap of pairwise Spearman's correlations between A) the shortlisted variables from Set 1 with k-means assigned cluster (phenotypic subtypes) in Set V1 horses (n=196); B) significant clinical variables identified in Set V2 (n=196) and k-means assigned cluster (phenotypic subtype). Stronger positive correlations between variables are increasingly red with stronger negative correlations between variables increasingly blue. In both sets, phenotype subtype 2 (Cluster 2) was not strongly associated with any variables, except for repeated ER episodes in Set V2.

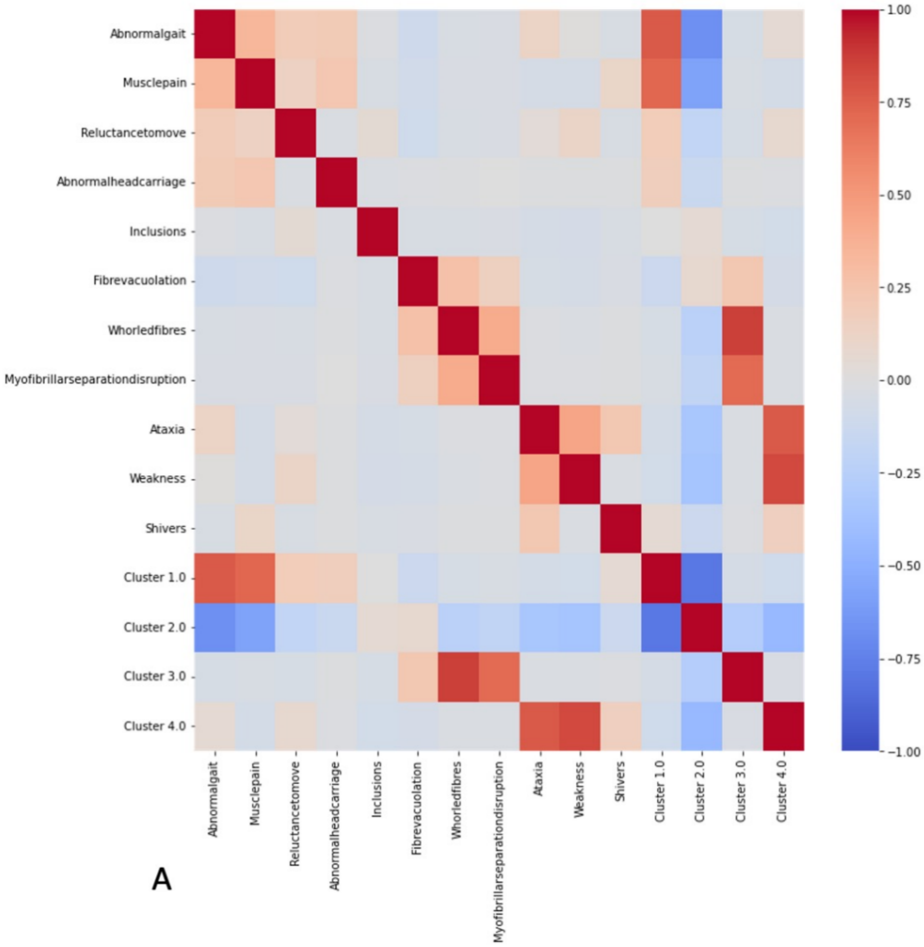
Abnormalgait
Musclepain
Reluctancetomove
Abnormalheadcarriage
Inclusions
Fibrevacuolation
Whorledfibres
Myofibrillarseparationdisruption
Ataxia
Weakness
Shivers
Cluster 1.0
Cluster 2.0
Cluster 3.0
Cluster 4.0
1.00
0.75
0.50
0.25
0.00
−0.25
−0.50
−0.75
−1.00

A

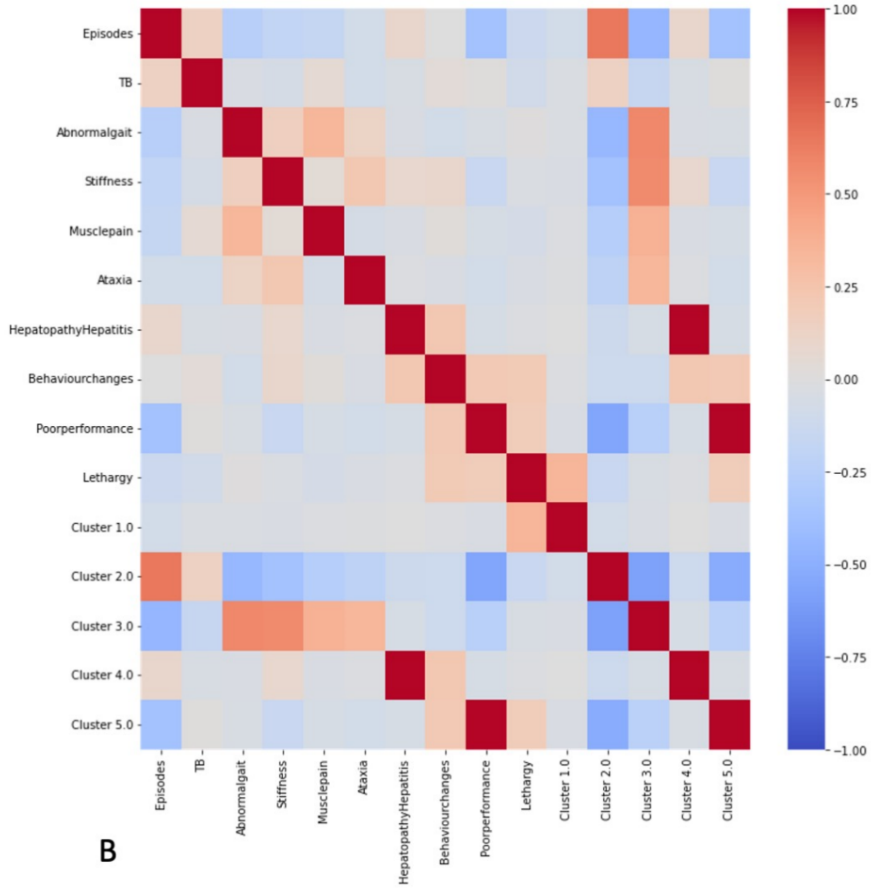
Episodes
TB
Abnormalgait
Stiffness
Musclepain
Ataxia
HepatopathyHepatitis
Behaviourchanges
Poorperformance
Lethargy
Cluster 1.0
Cluster 2.0
Cluster 3.0
Cluster 4.0
Cluster 5.0
1.00
0.75
0.50
0.25
0.00
−0.25
−0.50
−0.75
−1.00

B
