# Supplementary material for: Characterisation of phenotypic patterns in equine exercise‐associated myopathies
Source: Equine Vet J. 2024 Jul 5;57(2):347–61. doi: 10.1111/evj.14128 (PMC11807944; doi:10.1111/evj.14128)

Equine Veterinary Journal BEVA

Figure S7: Heatmap of pairwise Spearman's correlations between clinical variables significant between classic RER and combined non-classic EAMS subtypes from the Set 1 analysis, and both Set V1 and Set V2 validation analyses, and the three sets of classic RER and non-classic EAMS subtype groups. The classic RER subtype was the same as phenotypic subtype 1 in the Set 1 analysis, and represented phenotypic subtype 2 in both Set V1 and Set V2. The combined non-classic EAMS subtype in the Set 1 analysis consisted of phenotypic subtypes 2, 3 and 4, whilst in Set V1 it consisted of phenotypic subtypes 1, 3 and 4, and in Set V2 it consisted of phenotypic subtypes 1, 3, 4 and 5. Stronger positive correlations between variables are increasingly red with stronger negative correlations between variables increasingly blue. Classic RER in all 3 sets correlated positively, whilst non-classic EAMS in all sets correlated positively, with classic RER and non-classic EAMS correlating negatively.

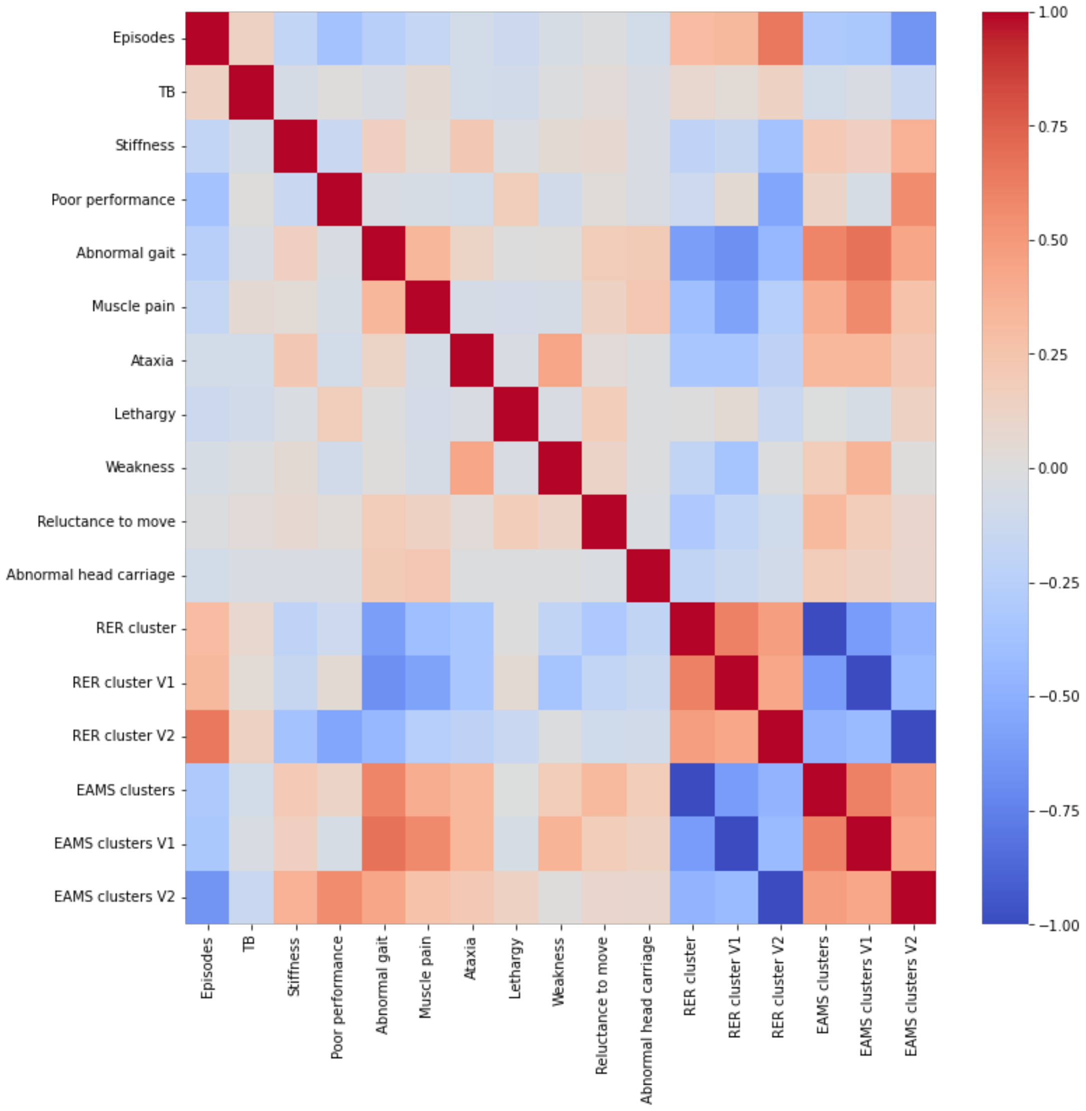

Supplement: Supplementary file 7 — Figure S7. Heatmap of pairwise Spearman's correlations between clinical variables significant between classic RER and combined non‐classic EAMS subtypes from the Set 1 analysis. [file EVJ-57-347-s018.pdf]
