# Supplementary material for: Characterisation of phenotypic patterns in equine exercise‐associated myopathies
Source: Equine Vet J. 2024 Jul 5;57(2):347–61. doi: 10.1111/evj.14128 (PMC11807944; doi:10.1111/evj.14128)

Equine Veterinary Journal BEVA

Figure S8: Violin plots showing difference in: distribution of binned serum CK activity scores between k-means assigned clusters (phenotypic subtype) in A) 98 horses from Set V1; and B) 99 horses from Set V2; binned serum CK activity scores between classic RER and non-classic EAMS subtype clusters in C) 98 horses from Set V1; and D) 99 horses from Set V2. The classic RER subtype was the same as phenotypic subtype 2 in both Set V1 and Set V2. The non-classic EAMS in Set V1 consisted of phenotypic subtypes 1, 3 and 4, and in Set V2 it consisted of phenotypic subtypes 1, 3, 4 and 5. Individual datapoints are shown in grey. There were no significant differences in CK activity score in Set V1, but in Set V2 the classic RER phenotype group had significantly higher CK activity scores than non-classic EAMS (p=0.017).

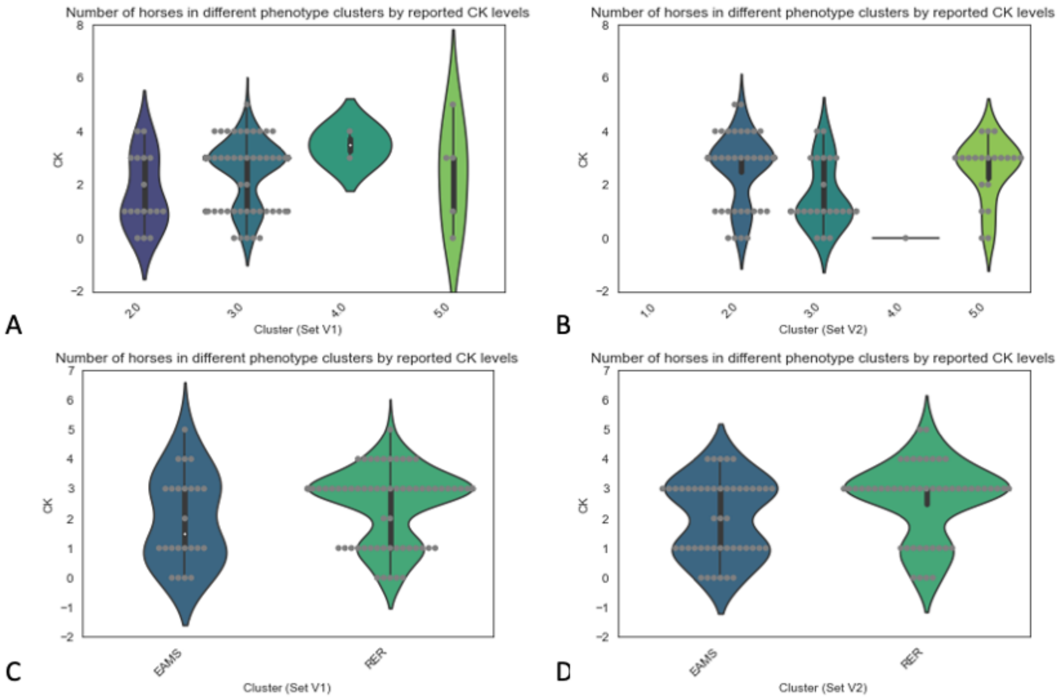

Supplement: Supplementary file 8 — Figure S8. Violin plots showing difference in: distribution of binned serum CK activity scores between k‐means assigned clusters (phenotypic subtype). [file EVJ-57-347-s004.pdf]
