# Supplementary material for: Characterisation of phenotypic patterns in equine exercise‐associated myopathies
Source: Equine Vet J. 2024 Jul 5;57(2):347–61. doi: 10.1111/evj.14128 (PMC11807944; doi:10.1111/evj.14128)

Equine Veterinary Journal BEVA

Figure S9: Vertical: hierarchical clustering using Ward's method of 109 Set 1 RER horses based on histological and clinical variables. Horizontal: hierarchical clustering using Ward's method of 45 clinical and histological variables based on 109 RER horses. Centre: heatmap showing the value for each variable for each horse, with 0 being blue and 1 being red. K-means assigned RER subtype is indicated on the left, with blue indicating non-classic EAMS and red classic RER subtype for each horse. The green circle highlights the cluster of clinical variables identified as a pattern of clinical signs in our previous analyses. The classic RER subtype was the same as phenotypic subtype 1 in the initial analysis. The non-classic EAMS subtype in the initial analysis consisted of phenotypic subtypes 2, 3 and 4.

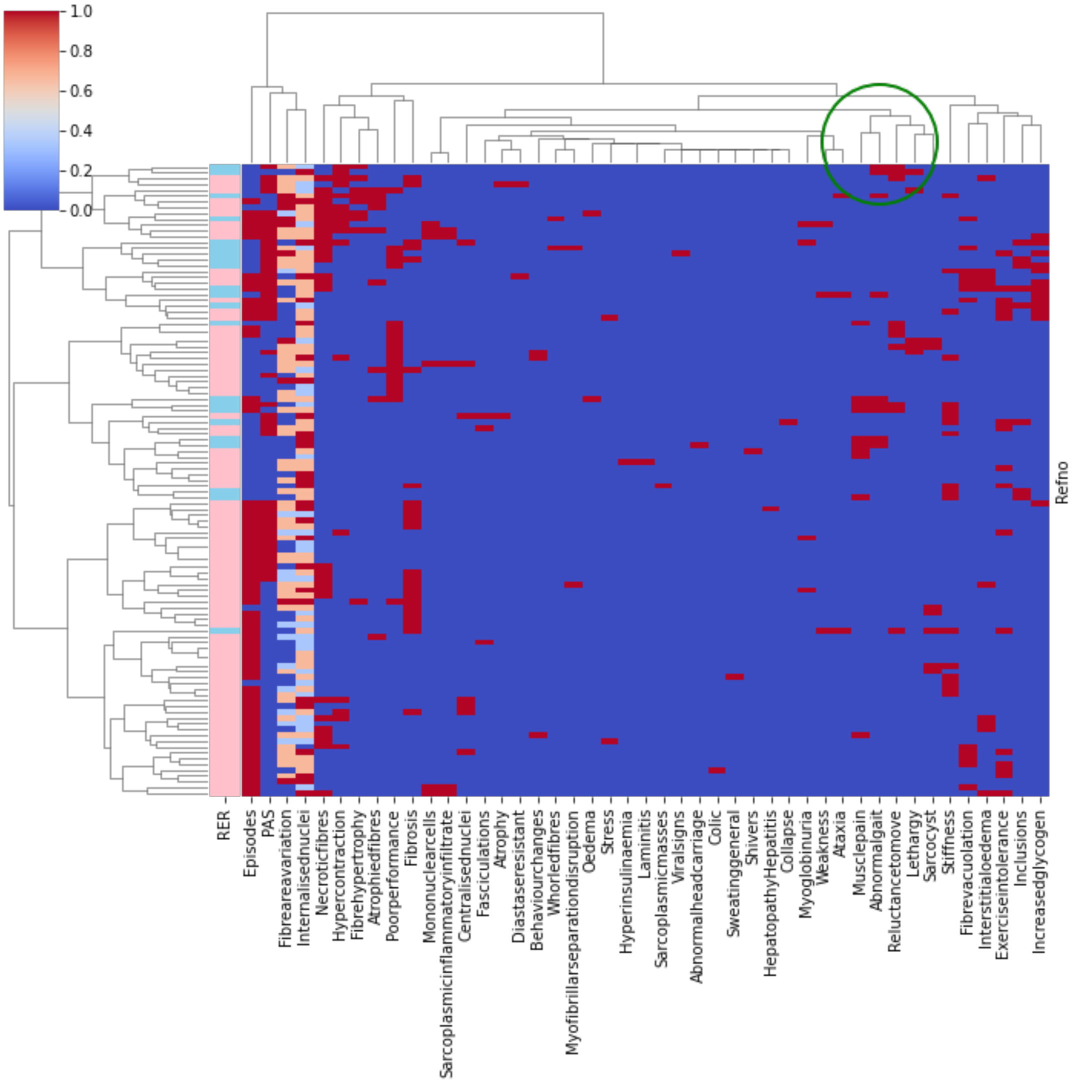

Supplement: Supplementary file 9 — Figure S9. Vertical: hierarchical clustering using Ward's method of 109 Set 1 RER horses based on histological and clinical variables. Horizontal: hierarchical clustering using Ward's method of 45 clinical and histological variables based on 109 RER horses. [file EVJ-57-347-s012.pdf]
