# Supplementary material for: Characterisation of phenotypic patterns in equine exercise‐associated myopathies
Source: Equine Vet J. 2024 Jul 5;57(2):347–61. doi: 10.1111/evj.14128 (PMC11807944; doi:10.1111/evj.14128)
Supplement: Supplementary file 10 — Figure S10. (A) Hierarchical clustering in Set 1; (B) Hierarchical clustering in Set 2. [file EVJ-57-347-s017.pdf]

Figure S10: A) Hierarchical clustering in Set 1; B) Hierarchical clustering in Set 2. In each plot, the vertical clustering is hierarchical clustering of horses based on clinical variables, and horizontal is hierarchical clustering of clinical variables. K-means assigned classic RER subtype from each set is indicated on the left of each graph. The green circle highlights the cluster of clinical variables identified as a pattern of clinical signs in our previous analyses. In Set V1, ataxia and weakness were clustered, whilst in Set V2 gait abnormalities, muscle pain, reluctance to move, stiffness exercise intolerance and poor performance clustered.

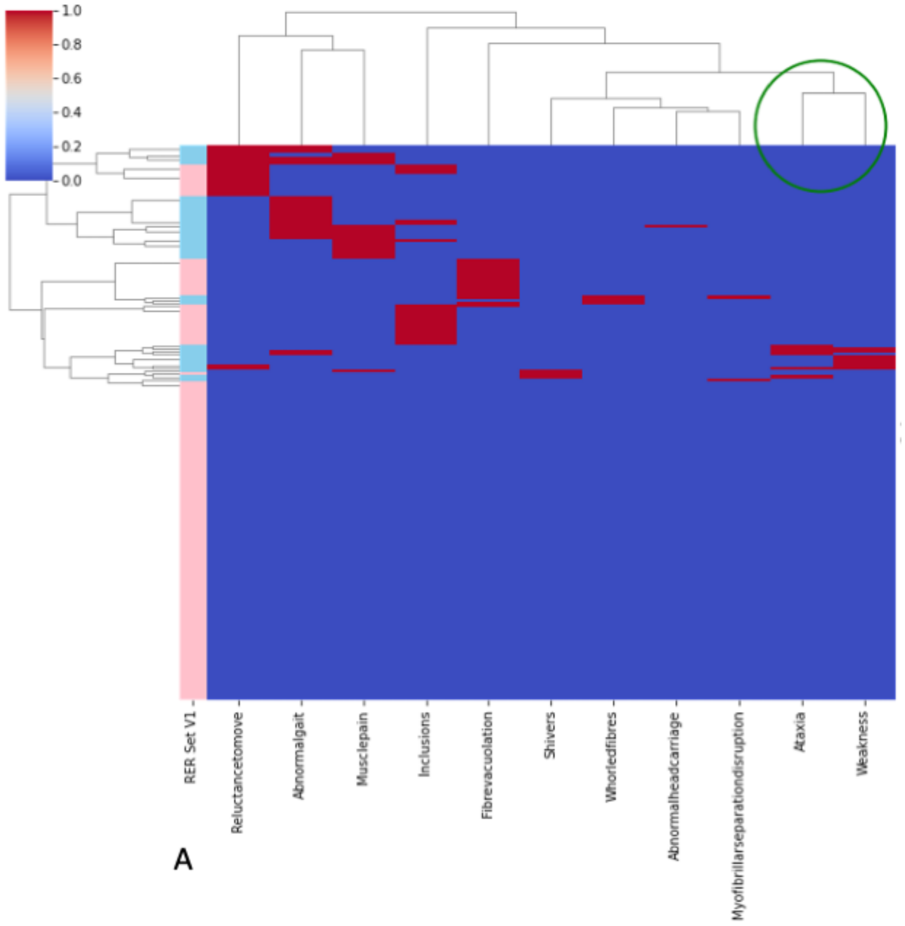

1.0
0.8
0.6
0.4
0.2
0.0
RER Set V1
Reluctancetomove
Abnormalgait
Musclepain
Inclusions
Fibrevacuolation
Shivers
Whorledfibres
Abnormalheadcarriage
Myofibrillarseparationdisruption
Ataxia
Weakness

A

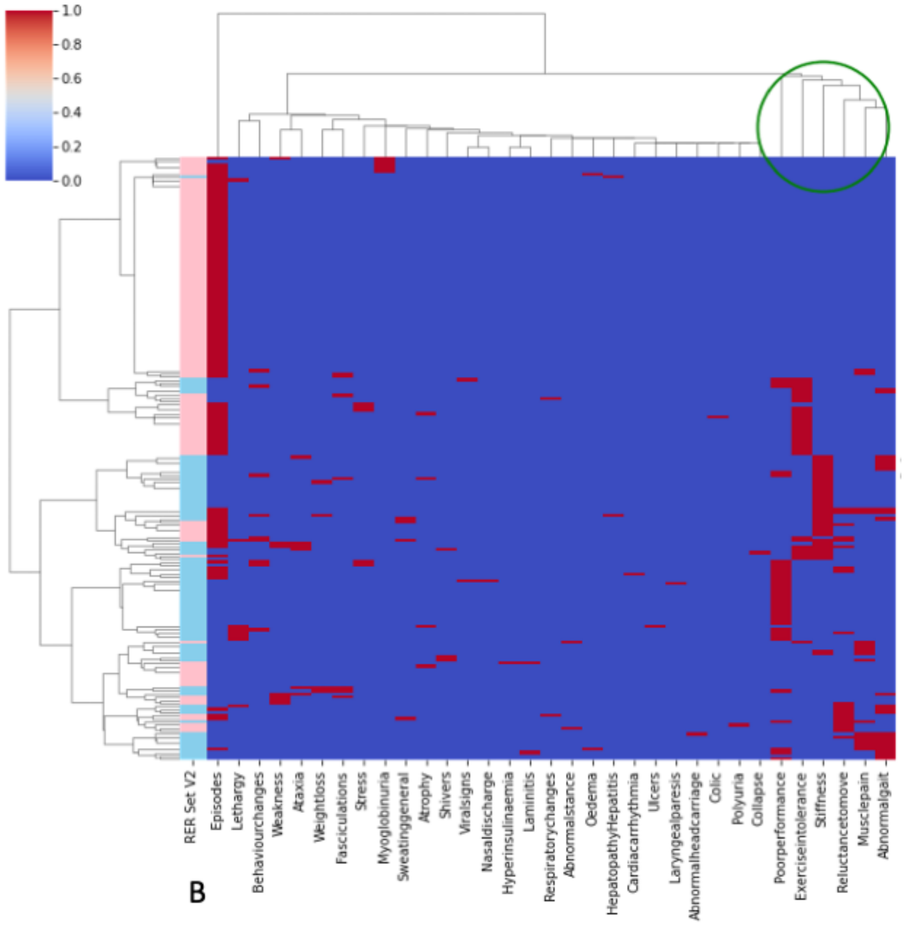

1.0
0.8
0.6
0.4
0.2
0.0
RER Set V2
Episodes
Lethargy
Behaviourchanges
Weakness
Ataxia
Weightloss
Fasciculations
Stress
Myoglobinuria
Sweatinggeneral
Atrophy
Shivers
Viralsigns
Nasaldischarge
Hyperinsulinaemia
Laminitis
Respiratorychanges
Abnormalstance
Oedema
HepatopathyHepatitis
Cardiacarrhythmia
Ulcers
Laryngealparesis
Abnormalheadcarriage
Colic
Polyuria
Collapse
Poorperformance
Exerciseintolerance
Stiffness
Reluctancetomove
Musclepain
Abnormalgait

B
