# Supplementary figures and images for: Characterisation of phenotypic patterns in equine exercise‐associated myopathies
Source: Equine Vet J. 2024 Jul 5;57(2):347–61. doi: 10.1111/evj.14128 (PMC11807944; doi:10.1111/evj.14128)

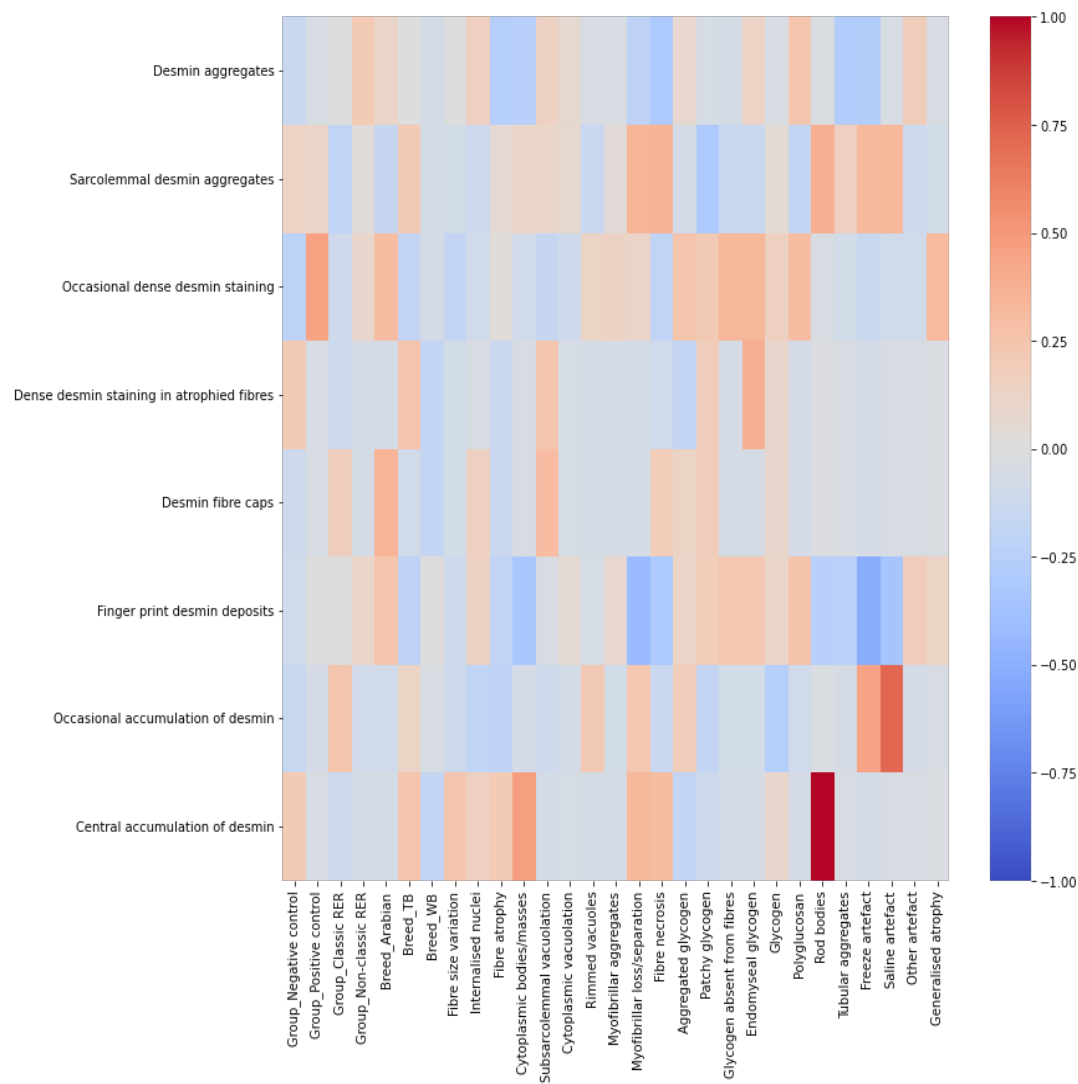

Supplement: Supplementary file 11 — Figure S11. Heatmap of Spearman's correlations of desmin‐related variables with selected signalment and histological variables from the histological comparison. [file EVJ-57-347-s010.pdf]
