# Supplementary material for: Characterisation of phenotypic patterns in equine exercise‐associated myopathies
Source: Equine Vet J. 2024 Jul 5;57(2):347–61. doi: 10.1111/evj.14128 (PMC11807944; doi:10.1111/evj.14128)
Supplement: Supplementary file 12 — Figure S12. Bar plot of significant variables in the final models. [file EVJ-57-347-s003.pdf]

Figure S12: Bar plot of significant variables in the final models. A) Significant binary variables remaining in a multinomial logistic regression model between the four disease groups, with the classic RER subtype as the reference category and breed accounted for in the model; B) significant ordinal variables remaining in the same multinomial logistic regression model as in A; C) glycogen score, the only significant variable in the logistic regression model between classic RER and non-classic EAMS, when breed was not accounted for in the model; D) significant binary variables remaining in a multinomial logistic regression model between the four disease groups, with the classic RER subtype as the reference category and breed accounted for in the model, when only Warmbloods and Arabians were included; E) significant ordinal variables remaining in the same multinomial logistic regression model as in D; F) degree of internalised nuclei, the only significant variable in the logistic regression model between classic RER and non-classic EAMS, when breed was accounted for in the model and only Warmbloods and Arabians were included. Error bars represent standard deviation.

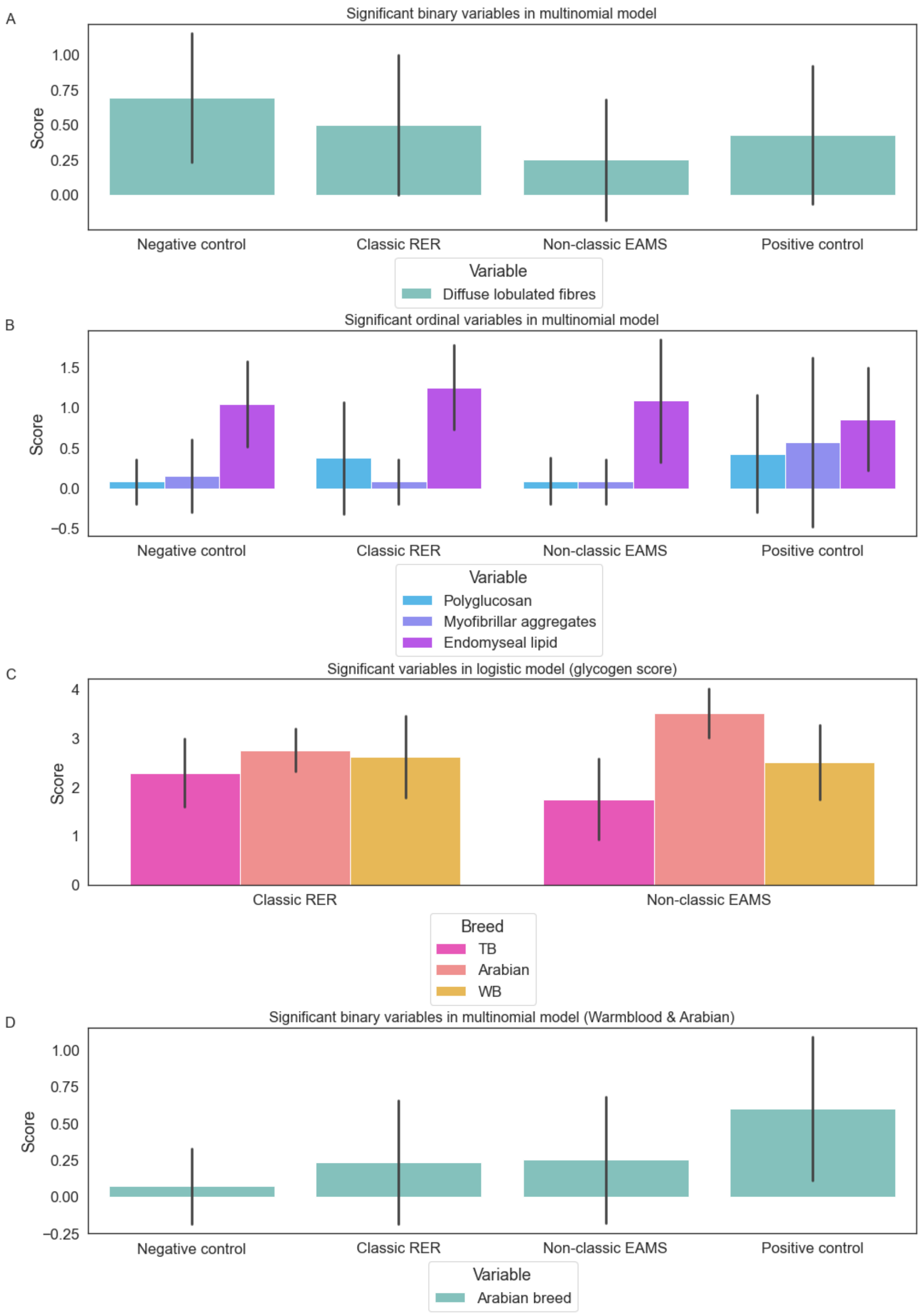
A
Significant binary variables in multinomial model
Score
1.00
0.75
0.50
0.25
0.00
Negative control
Classic RER
Non-classic EAMS
Positive control
Variable
Diffuse lobulated fibres
B
Significant ordinal variables in multinomial model
Score
1.5
1.0
0.5
0.0
−0.5
Negative control
Classic RER
Non-classic EAMS
Positive control
Variable
Polyglucosan
Myofibrillar aggregates
Endomyseal lipid
C
Significant variables in logistic model (glycogen score)
Score
4
3
2
1
0
Classic RER
Non-classic EAMS
Breed
TB
Arabian
WB
D
Significant binary variables in multinomial model (Warmblood & Arabian)
Score
1.00
0.75
0.50
0.25
0.00
−0.25
Negative control
Classic RER
Non-classic EAMS
Positive control
Variable
Arabian breed

E

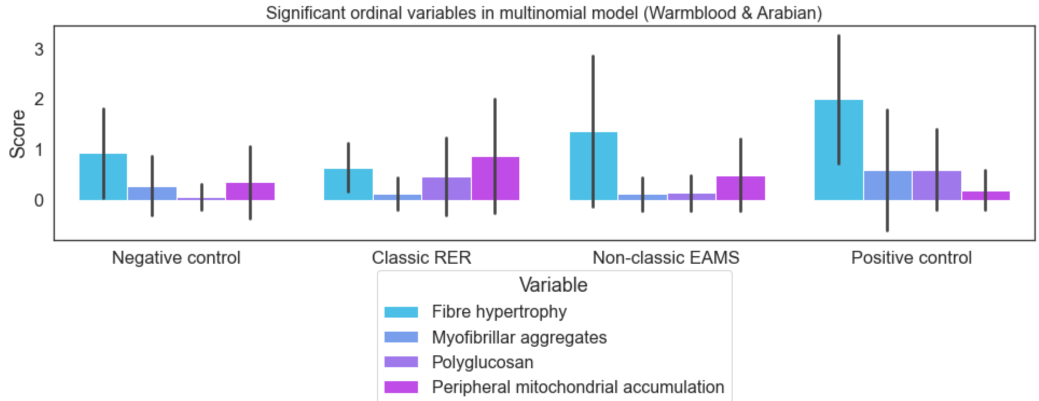
Significant ordinal variables in multinomial model (Warmblood & Arabian)
Score
3
2
1
0
Negative control
Classic RER
Non-classic EAMS
Positive control
Variable
Fibre hypertrophy
Myofibrillar aggregates
Polyglucosan
Peripheral mitochondrial accumulation

F

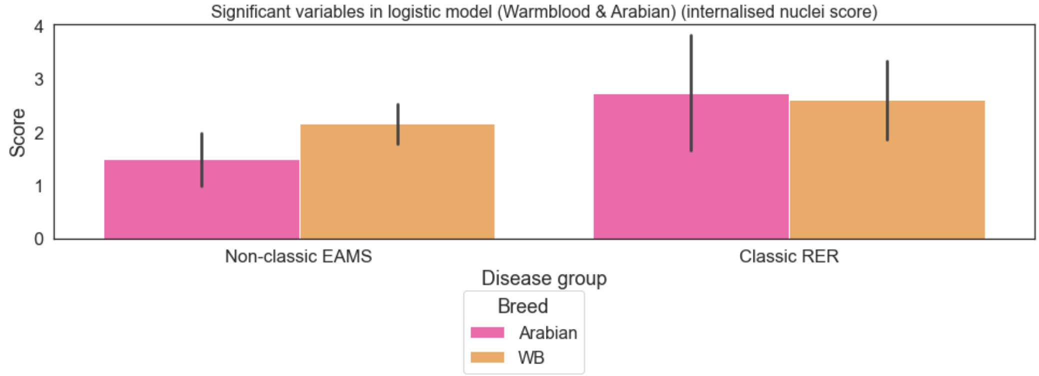
Significant variables in logistic model (Warmblood & Arabian) (internalised nuclei score)
Score
4
3
2
1
0
Non-classic EAMS
Classic RER
Disease group
Breed
Arabian
WB
