# Supplementary material for: Characterisation of phenotypic patterns in equine exercise‐associated myopathies
Source: Equine Vet J. 2024 Jul 5;57(2):347–61. doi: 10.1111/evj.14128 (PMC11807944; doi:10.1111/evj.14128)
Supplement: Supplementary file 13 — Figure S13. Biplots of PCA scores and the loadings of variables with loadings >0.2 or <−0.2 on either of the plotted principal components. [file EVJ-57-347-s013.pdf]

Equine Veterinary Journal BEVA

Figure S13:

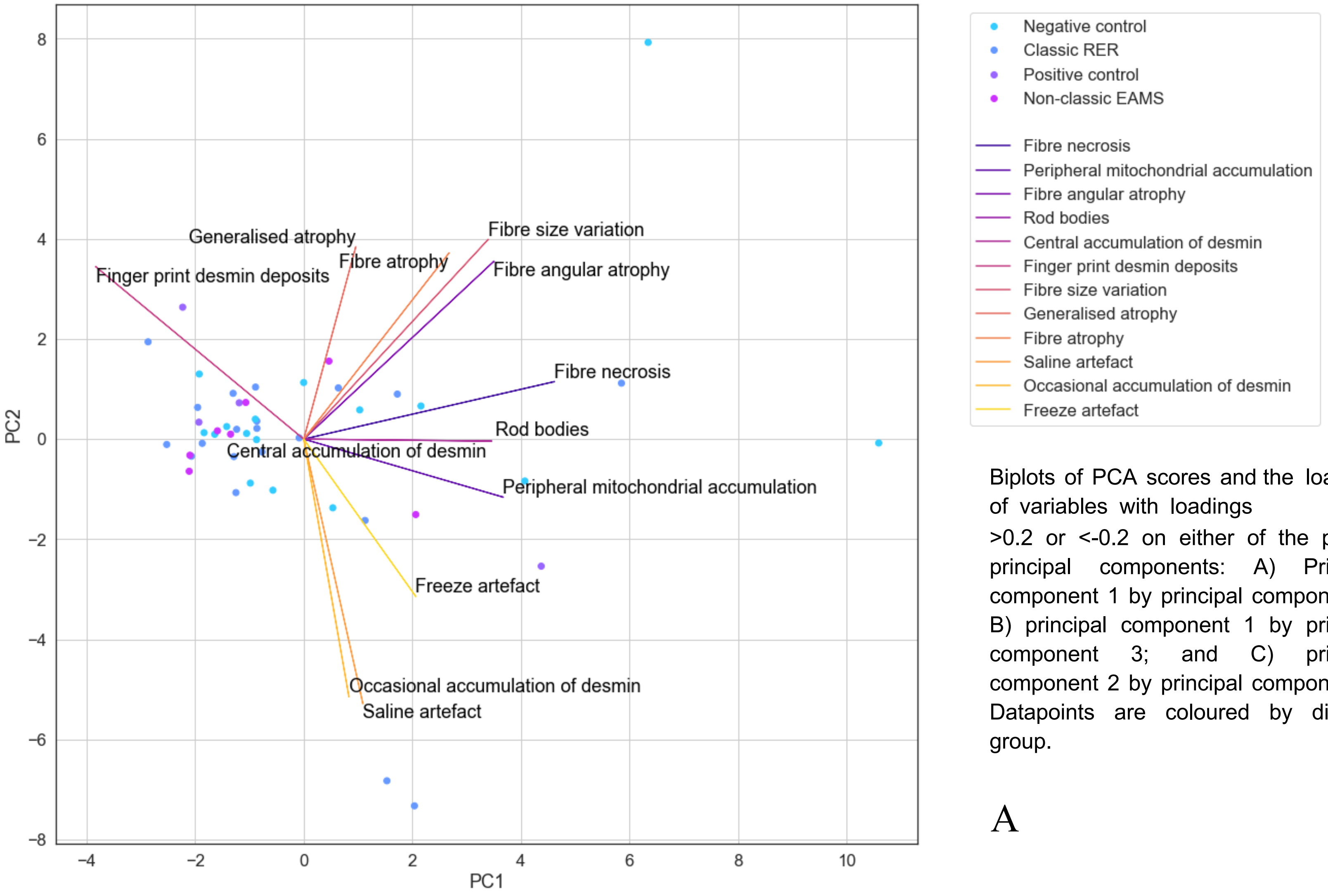

Biplots of PCA scores and the loadings of variables with loadings >0.2 or <-0.2 on either of the plotted principal components: A) Principal component 1 by principal component 2; B) principal component 1 by principal component 3; and C) principal component 2 by principal component 3. Datapoints are coloured by disease group.

A

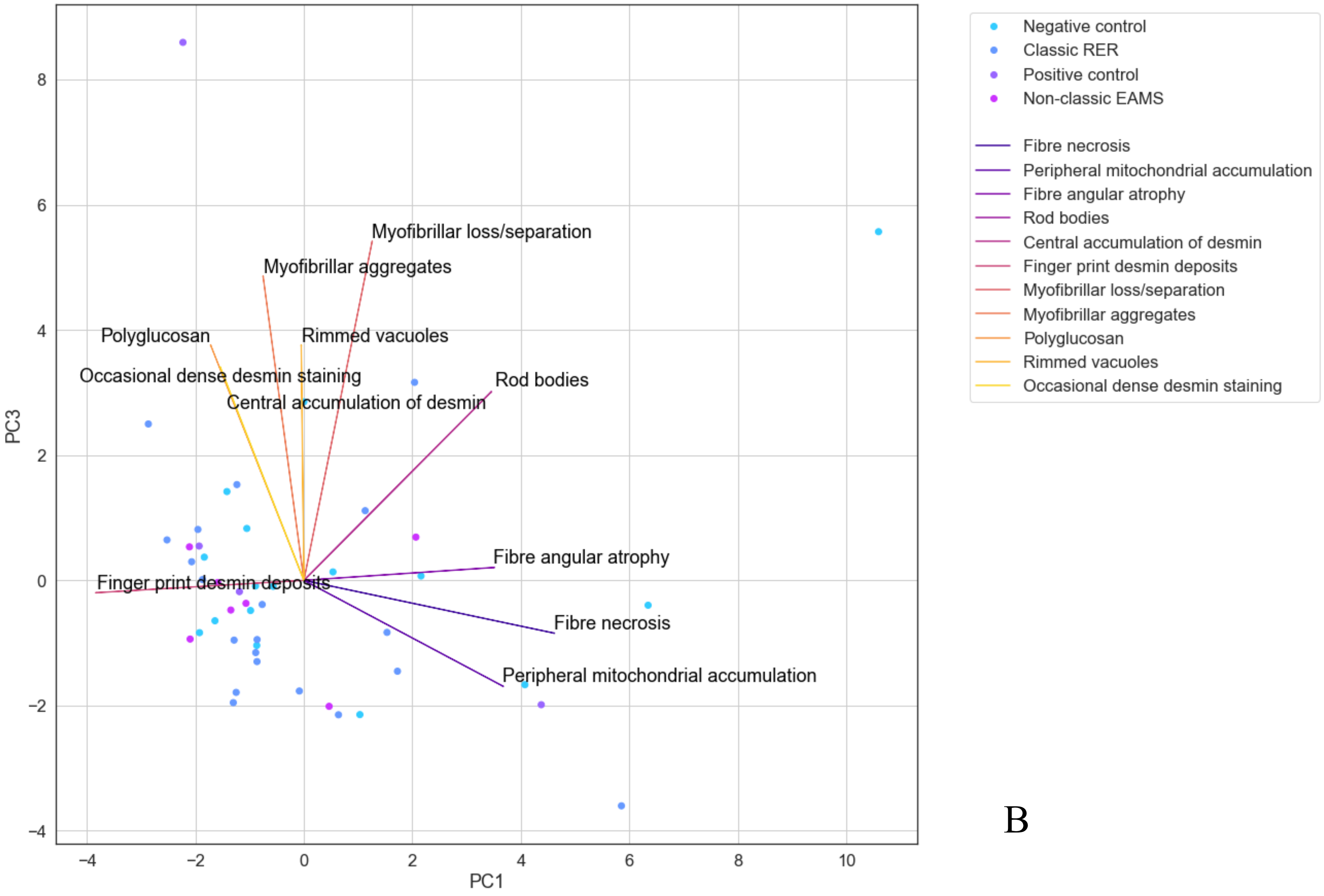
PC3
PC1
−4
−2
0
2
4
6
8
10
Myofibrillar loss/separation
Myofibrillar aggregates
Polyglucosan
Rimmed vacuoles
Occasional dense desmin staining
Central accumulation of desmin
Rod bodies
Fibre angular atrophy
Finger print desmin deposits
Fibre necrosis
Peripheral mitochondrial accumulation
Negative control
Classic RER
Positive control
Non-classic EAMS
Fibre necrosis
Peripheral mitochondrial accumulation
Fibre angular atrophy
Rod bodies
Central accumulation of desmin
Finger print desmin deposits
Myofibrillar loss/separation
Myofibrillar aggregates
Polyglucosan
Rimmed vacuoles
Occasional dense desmin staining

B

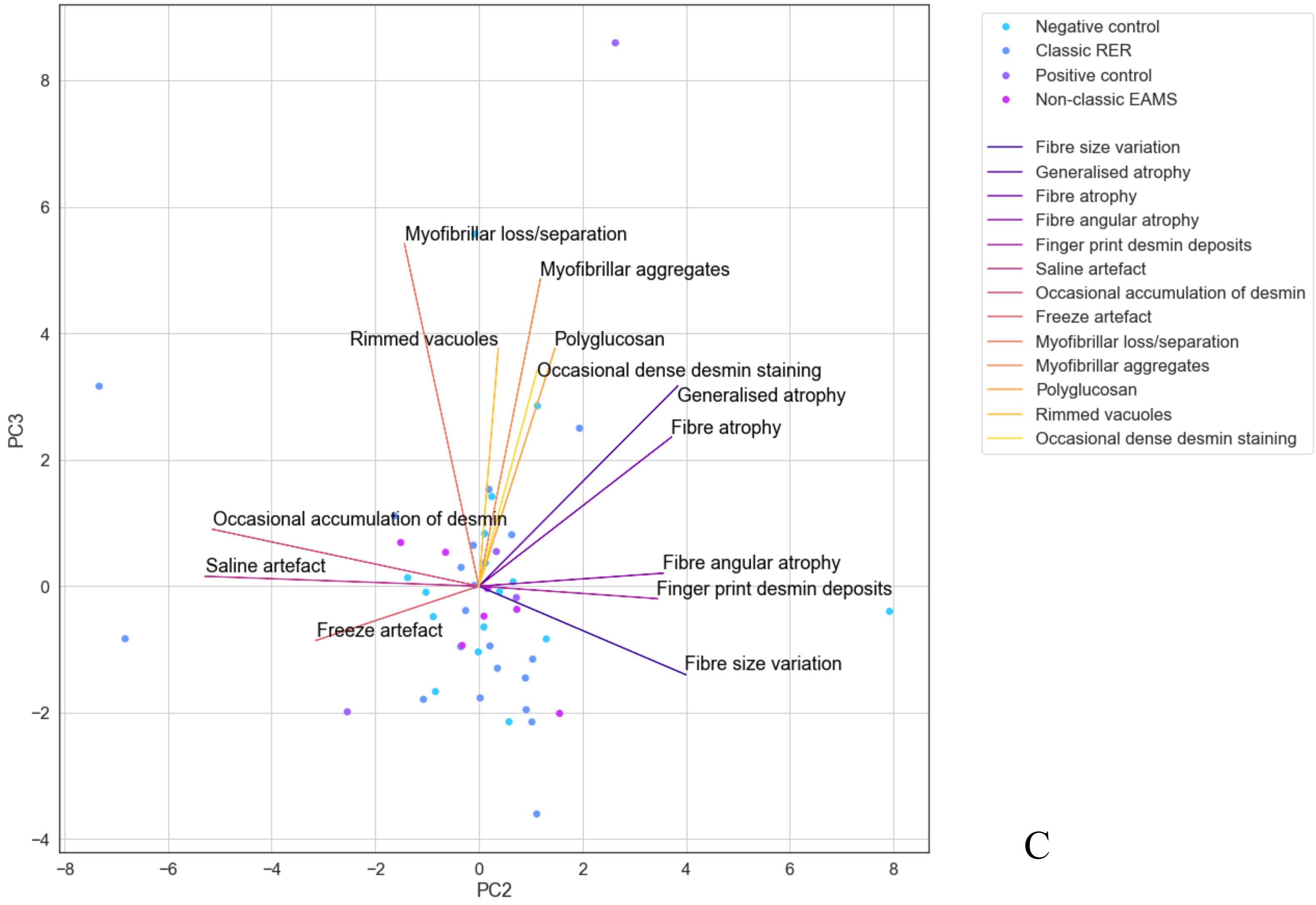
Negative control
Classic RER
Positive control
Non-classic EAMS
Fibre size variation
Generalised atrophy
Fibre atrophy
Fibre angular atrophy
Finger print desmin deposits
Saline artefact
Occasional accumulation of desmin
Freeze artefact
Myofibrillar loss/separation
Myofibrillar aggregates
Polyglucosan
Rimmed vacuoles
Occasional dense desmin staining
PC3
PC2
−8
−6
−4
−2
0
2
4
6
8

C
