# Supplementary material for: Characterisation of phenotypic patterns in equine exercise‐associated myopathies
Source: Equine Vet J. 2024 Jul 5;57(2):347–61. doi: 10.1111/evj.14128 (PMC11807944; doi:10.1111/evj.14128)
Supplement: Supplementary file 14 — Table S1. Signalment, clinical history, biochemistry, environment and histological variables recorded for analysis of the Comparative Neuromuscular Diseases Laboratory diagnostic service records. [file EVJ-57-347-s007.pdf]

**Table S1:** Signalment, clinical history, biochemistry, environment and histological variables recorded for analysis of the Comparative Neuromuscular Diseases Laboratory diagnostic service records.

| Variable type | Variable | How was variable recorded | Percentage of horses with missing data | | Was variable included in ER phenotyping analyses? | |
|---|---|---|---|---|---|---|
| | | | Total data | Exercise-associated myopathy horses | Clustering | Association |
| Signalment | Year | Year sample was submitted | 0% | 0 | No | No |
| Signalment | Age | Horse age at submission (integer) | 9.58% | 4.82% | No | Yes |
| Signalment | Sex | Female – 0; Male - 1 | 3.47% | 1.38% | No | Yes |
| Signalment | Breed type | Categorical: Arabian; Connemara; Cob; Cross; Irish; Native pony; Other; Thoroughbred (TB); Standardbred (STB); Warmblood (WB) | 8.58% | 4.12% | No | Yes |
| Signalment | Diagnosis type | Categorical: Atypical myopathy; Centronuclear myopathy; Equine motor neurone disease (EMND); Idiopathic myopathy; Idiopathic neuropathy; Inconclusive; Mitochondrial myopathy; Myositis; Neurogenic atrophy; No neuromuscular disease; Other myopathies; Other neuropathies; PSSM1; PSSM1 negative test only; PSSM2; RER; Sarcocystosis; Vacuolar myopathy | 0% | 0% | No | No |
| Environment | Use | Categorical: Competition; Jumping; Eventing; | 92.47% | 91.03% | No | No |

| | | | | | | |
|---|---|---|---|---|---|---|
| | | Hunting; Retired; Youngstock; Polo; Dressage; Breeding; Harness racing; Racing; Leisure; Riding club/ Pony Club (RC/PC); Endurance; Driving; Off work | | | | |
| Environment | Fitness | Ordinal scale from 0 (not in work) to 4 (High) | 94.80% | 95.86% | No | No |
| Environment | Low carb, high fat diet | Not applicable (0); applicable (1) | 99.00% | 97.93% | No | No |
| Environment | Access to grazing | Not applicable (0); applicable (1) | 96.26% | 95.17% | No | No |
| Environment | Dantrolene medication | Not applicable (0); applicable (1) | 95.80% | 96.86% | No | No |
| Environment | Recent dietary changes | Not applicable (0); applicable (1) | 96.26% | 96.86% | No | No |
| Clinical history | Death | Not reported (0); reported (1) | 14.78% | 2.76% | No | No |
| Clinical history | Bradycardia | Not reported (0); reported (1) | 14.78% | 2.76% | No | No |
| Clinical history | Myoglobinuria | Not reported (0); reported (1) | 14.78% | 2.76% | Yes | Yes |
| Clinical history | Polyuria | Not reported (0); reported (1) | 14.78% | 2.76% | Yes | Yes |
| Clinical history | Polydipsia | Not reported (0); reported (1) | 14.78% | 2.76% | No | No |
| Clinical history | Renal failure | Not reported (0); reported (1) | 14.78% | 2.76% | No | No |
| Clinical history | Hepatopathy or hepatitis | Not reported (0); reported (1) | 14.78% | 2.76% | Yes | Yes |
| Clinical history | Hyperinsulina-emia | Not reported (0); reported (1) | 14.78% | 2.76% | Yes | Yes |
| Clinical history | Muscle pain | Not reported (0); reported (1) | 14.78% | 2.76% | Yes | Yes |
| Clinical history | Collapse | Not reported (0); reported (1) | 14.78% | 2.76% | Yes | Yes |
| Clinical history | Exercise intolerance | Not reported (0); reported (1) | 14.78% | 2.76% | Yes | Yes |
| Clinical history | Reluctance to go forward | Not reported (0); reported (1) | 14.78% | 2.76% | Yes | Yes |
| Clinical history | Poor performance | Not reported (0); reported (1) | 14.78% | 2.76% | Yes | Yes |
| Clinical history | Difficulty backing up | Not reported (0); reported (1) | 14.78% | 2.76% | No | No |

| | | | | | | |
|---|---|---|---|---|---|---|
| Clinical history | Lethargy | Not reported (0); reported (1) | 14.78% | 2.76% | Yes | Yes |
| Clinical history | Muscle atrophy | Not reported (0); reported (1) | 14.78% | 2.76% | Yes | Yes |
| Clinical history | Muscle hypertrophy | Not reported (0); reported (1) | 14.78% | 2.76% | No | No |
| Clinical history | 'Shivers' | Not reported (0); reported (1) | 14.78% | 2.76% | Yes | Yes |
| Clinical history | 'Wobblers' | Not reported (0); reported (1) | 14.78% | 2.76% | No | No |
| Clinical history | Weakness | Not reported (0); reported (1) | 14.78% | 2.76% | Yes | Yes |
| Clinical history | Ataxia | Not reported (0); reported (1) | 14.78% | 2.76% | Yes | Yes |
| Clinical history | Lower motor neuron signs | Not reported (0); reported (1) | 14.78% | 2.76% | No | No |
| Clinical history | Incontinence | Not reported (0); reported (1) | 14.78% | 2.76% | No | No |
| Clinical history | Cranial nerve deficits | Not reported (0); reported (1) | 14.78% | 2.76% | No | No |
| Clinical history | Dysphagia | Not reported (0); reported (1) | 14.78% | 2.76% | No | No |
| Clinical history | 'Quidding' | Not reported (0); reported (1) | 14.78% | 2.76% | No | No |
| Clinical history | Recurrent laryngeal neuropathy | Not reported (0); reported (1) | 14.78% | 2.76% | No | No |
| Clinical history | Soft palate displacement | Not reported (0); reported (1) | 14.78% | 2.76% | No | No |
| Clinical history | Pharyngeal collapse | Not reported (0); reported (1) | 14.78% | 2.76% | No | No |
| Clinical history | Sweating – neck | Not reported (0); reported (1) | 14.78% | 2.76% | No | No |
| Clinical history | Sweating – generalised | Not reported (0); reported (1) | 14.78% | 2.76% | Yes | Yes |
| Clinical history | Sweating – hindquarters | Not reported (0); reported (1) | 14.78% | 2.76% | No | No |
| Clinical history | Sweating – ventral | Not reported (0); reported (1) | 14.78% | 2.76% | No | No |
| Clinical history | Colic | Not reported (0); reported (1) | 14.78% | 2.76% | Yes | Yes |
| Clinical history | Weight loss | Not reported (0); reported (1) | 14.78% | 2.76% | Yes | Yes |
| Clinical history | Stress behaviour | Not reported (0); reported (1) | 14.78% | 2.76% | Yes | Yes |
| Clinical history | Behavioural changes | Not reported (0); reported (1) | 14.78% | 2.76% | Yes | Yes |
| Clinical history | Appetite changes | Not reported (0); reported (1) | 14.78% | 2.76% | No | No |

| | | | | | | |
|---|---|---|---|---|---|---|
| Clinical history | Muscle fasciculations | Not reported (0); reported (1) | 14.78% | 2.76% | Yes | Yes |
| Clinical history | Stiffness | Not reported (0); reported (1) | 14.78% | 2.76% | Yes | Yes |
| Clinical history | Muscle oedema | Not reported (0); reported (1) | 14.78% | 2.76% | Yes | Yes |
| Clinical history | Abnormal gait | Not reported (0); reported (1) | 14.78% | 2.76% | Yes | Yes |
| Clinical history | Abnormal stance | Not reported (0); reported (1) | 14.78% | 2.76% | Yes | Yes |
| Clinical history | Abnormal head carriage | Not reported (0); reported (1) | 14.78% | 2.76% | Yes | Yes |
| Clinical history | High tail carriage | Not reported (0); reported (1) | 14.78% | 2.76% | No | No |
| Clinical history | Weight shifting | Not reported (0); reported (1) | 14.78% | 2.76% | No | No |
| Clinical history | Recumbency | Not reported (0); reported (1) | 14.78% | 2.76% | No | No |
| Clinical history | Retinitis or retinopathy | Not reported (0); reported (1) | 14.78% | 2.76% | No | No |
| Clinical history | Dilated pupils | Not reported (0); reported (1) | 14.78% | 2.76% | No | No |
| Clinical history | Laryngeal paresis | Not reported (0); reported (1) | 14.78% | 2.76% | No | No |
| Clinical history | Respiratory changes | Not reported (0); reported (1) | 14.78% | 2.76% | Yes | Yes |
| Clinical history | Tail paresis | Not reported (0); reported (1) | 14.78% | 2.76% | No | No |
| Clinical history | Poor rectal or anal tone | Not reported (0); reported (1) | 14.78% | 2.76% | No | No |
| Clinical history | Faecal retention | Not reported (0); reported (1) | 14.78% | 2.76% | No | No |
| Clinical history | Loss of sensation | Not reported (0); reported (1) | 14.78% | 2.76% | No | No |
| Clinical history | Fever | Not reported (0); reported (1) | 14.78% | 2.76% | No | No |
| Clinical history | Discospondy-litis | Not reported (0); reported (1) | 14.78% | 2.76% | No | No |
| Clinical history | Vasculitis | Not reported (0); reported (1) | 14.78% | 2.76% | No | No |
| Clinical history | Laminitis | Not reported (0); reported (1) | 14.78% | 2.76% | Yes | Yes |
| Clinical history | Viral signs | Not reported (0); reported (1) | 14.78% | 2.76% | Yes | Yes |
| Clinical history | Steatitis | Not reported (0); reported (1) | 14.78% | 2.76% | No | No |
| Clinical history | Granulomas | Not reported (0); reported (1) | 14.78% | 2.76% | No | No |

| | | | | | | |
|---|---|---|---|---|---|---|
| Clinical history | Lipoma | Not reported (0); reported (1) | 14.78% | 2.76% | No | No |
| Clinical history | EHV positive | Not reported (0); reported (1) | 14.78% | 2.76% | No | No |
| Clinical history | Sarcoids | Not reported (0); reported (1) | 14.78% | 2.76% | No | No |
| Clinical history | Pituitary adenoma | Not reported (0); reported (1) | 14.78% | 2.76% | No | No |
| Clinical history | Urticaria | Not reported (0); reported (1) | 14.78% | 2.76% | No | No |
| Clinical history | Central blindness | Not reported (0); reported (1) | 14.78% | 2.76% | No | No |
| Clinical history | Seizures | Not reported (0); reported (1) | 14.78% | 2.76% | No | No |
| Clinical history | 'Wind-sucking' | Not reported (0); reported (1) | 14.78% | 2.76% | No | No |
| Clinical history | Ulcers | Not reported (0); reported (1) | 14.78% | 2.76% | Yes | Yes |
| Clinical history | Cardiac arrythmia | Not reported (0); reported (1) | 14.78% | 2.76% | No | No |
| Clinical history | Diarrhoea | Not reported (0); reported (1) | 14.78% | 2.76% | No | No |
| Clinical history | Nasal discharge | Not reported (0); reported (1) | 14.78% | 2.76% | Yes | Yes |
| Clinical history | Chronic cough | Not reported (0); reported (1) | 14.78% | 2.76% | No | No |
| Clinical history | Chorioptic mange | Not reported (0); reported (1) | 14.78% | 2.76% | No | No |
| Clinical history | Cryptorchid | Not reported (0); reported (1) | 14.78% | 2.76% | No | No |
| Clinical history | High faecal worm egg count | Not reported (0); reported (1) | 14.78% | 2.76% | No | No |
| Clinical history | PSSM1 genotype | PSSM1 negative (0); PSSM1 positive (1) | 50.73% | 66.21% | No | No |
| Clinical history | Malignant hyperthermia (MH) genotype | MH negative (0); MH positive (1) | 99.64% | 100% | No | No |
| Clinical history | Hyperkalaem-ic periodic paralysis (HYPP) genotype | HYPP negative (0); HYPP positive (1) | 99.91% | 100% | No | No |
| Clinical history | Recurrent ER episodes | Not reported (0); reported (1) | 14.78% | 2.76% | Yes | Yes |
| Clinical history | X-ray results | Reported normal (0); reported abnormal (1) | 99.91% | 100% | No | No |

| | | | | | | |
|---|---|---|---|---|---|---|
| Clinical history | IRU scintigraphy results | Reported normal (0); reported abnormal (1) | 98.72% | 98.62% | No | No |
| Clinical history | Electromyogr-aphy results | Reported normal (0); reported abnormal (1) | 99.54% | 100% | No | No |
| Biochemistry | Creatine kinase (CK) activity | Ordinal scale: 0 (<700 U/L); 1 (700-1,000 U/L); 2 (1,000-3,000 U/L); 3 (3,000-10,000 U/L); 4 (10,000 – 20,000 U/L); 5 (>20,000 U/L) | 63.41% | 48.97% | No | No |
| Biochemistry | Aspartate aminotransfer-ase (AST) activity | Ordinal scale: 0 (<420 U/L); 1 (420-750 U/L); 2 (750-1,000 U/L); 3 (1,000-2,500 U/L); 4 (2,500 – 5,000 U/L); 5 (>5,000 U/L) | 66.97% | 53.79% | No | No |
| Biochemistry | Lactate dehydrogenase (LDH) | Within normal range (0); Abnormal (1) | 97.26% | 97.93% | No | No |
| Biochemistry | Gamma glutamyltrans-ferase (GGT) | Within normal range (0); Abnormal (1) | 97.35% | 97.93% | No | No |
| Biochemistry | Anaemia | Not present (0); present (1) | 97.35% | 97.93% | No | No |
| Biochemistry | Leucocytosis | Not present (0); present (1) | 97.17% | 100% | No | No |
| Biochemistry | Leucopoenia | Not present (0); present (1) | 97.35% | 100% | No | No |
| Biochemistry | Hyperfibrinog-enaemia | Not present (0); present (1) | 97.26% | 97.93% | No | No |
| Biochemistry | Bilirubin | Within normal range (0); Abnormal (1) | 97.35% | 97.93% | No | No |
| Biochemistry | Vitamin E | Within normal range (0); Abnormal (1) | 97.17% | 97.93% | No | No |
| Biochemistry | (GSHPx) | Within normal range (0); Abnormal (1) | 98.91% | 97.93% | No | No |
| Biochemistry | Elevated white blood cell count | Not present (0); present (1) | 97.35% | 97.93% | No | No |
| Biochemistry | Neutropoenia | Not present (0); present (1) | 97.35% | 97.93% | No | No |
| Biochemistry | Low selenium | Not present (0); present (1) | 97.26% | 97.93% | No | No |
| Biochemistry | Serum amyloid A | Within normal range (0); Abnormal (1) | 99.64% | 100% | No | No |

| | | | | | | |
|---|---|---|---|---|---|---|
| Biochemistry | Hypercalcae-mia | Not present (0); present (1) | 99.45% | 100% | No | No |
| Biochemistry | Hyperkalaemia | Not present (0); present (1) | 97.35% | 97.93% | No | No |
| Biochemistry | Cerebrospinal fluid changes | Not present (0); present (1) | 99.91% | 100% | No | No |
| Biochemistry | Increased FC of Cl | Not present (0); present (1) | 99.91% | 100% | No | No |
| Biochemistry | Organic acid results | Within normal range (0); Abnormal (1) | 98.45% | 98.62% | No | No |
| Biochemistry | Acylcarnitine results | Within normal range (0); Abnormal (1) | 98.81% | 99.31% | No | No |
| Biochemistry | Triglyceride elevation | Not present (0); present (1) | 99.91% | 100% | No | No |
| Biochemistry | Triglyceride concentration | Continuous numerical data | 99.91% | 100% | No | No |
| Biochemistry | Pyruvate | Continuous numerical data | 99.82% | 99.31% | No | No |
| Biochemistry | Lactate | Continuous numerical data | 99.82% | 99.31% | No | No |
| Biochemistry | L:P ratio | Ratio | 99.82% | 99.31% | No | No |
| Histological | Histological disease stage | Ordinal scale: 1 (sub-acute); 2 (acute), 3 (acute-chronic); 4 (chronic) | 50.27% | 2.76% | No | Yes |
| Histological | Histological disease severity | Ordinal scale: normal (0); normal-mild (1); mild (2); mild-moderate (3); moderate (4); moderate-severe (5); severe (6) | 41.06% | 2.76% | No | Yes |
| Histological | Fibre area variation | Ordinal scale: not increased (0); slightly increased (1); moderately increased (2); moderately to highly increased (3); highly increased (4); excessively increased (5) | 30.57% | 0% | Yes | Yes |
| Histological | Internalised nuclei | Ordinal scale: not increased (0); slightly increased (1); moderately increased (2); moderately to highly increased (3); highly | 30.57% | 0% | Yes | Yes |

| | | | | | | |
|---|---|---|---|---|---|---|
| | | increased (4); excessively increased (5) | | | | |
| Histological | Centralised nuclei | Not present (0); present (1) | 30.57% | 0% | Yes | Yes |
| Histological | Angular atrophy | Not present (0); present (1) | 30.57% | 0% | No | No |
| Histological | Fibre hypertrophy | Not present (0); present (1) | 30.57% | 0% | Yes | Yes |
| Histological | Fibre hypercontract-ion | Not present (0); present (1) | 30.57% | 0% | Yes | Yes |
| Histological | Fibre atrophy | Not present (0); present (1) | 30.57% | 0% | Yes | Yes |
| Histological | Target fibres | Not present (0); present (1) | 30.57% | 0% | No | No |
| Histological | Fibre splitting | Not present (0); present (1) | 30.57% | 0% | No | No |
| Histological | Whorled fibres | Not present (0); present (1) | 30.57% | 0% | Yes | Yes |
| Histological | Inclusions | Not present (0); present (1) | 30.57% | 0% | Yes | Yes |
| Histological | Fibre vacuolation | Not present (0); present (1) | 30.57% | 0% | Yes | Yes |
| Histological | Cleft fibres | Not present (0); present (1) | 30.57% | 0% | No | No |
| Histological | Myofibrillar aggregates | Not present (0); present (1) | 30.57% | 0% | Yes | Yes |
| Histological | Myofibrillar separation and/or disruption | Not present (0); present (1) | 30.57% | 0% | Yes | Yes |
| Histological | Myofibril loss | Not present (0); present (1) | 30.57% | 0% | No | No |
| Histological | Sarcoplasmic masses | Not present (0); present (1) | 30.57% | 0% | Yes | Yes |
| Histological | Birefringence | Not present (0); present (1) | 30.57% | 0% | No | No |
| Histological | Fibrosis | Not present (0); present (1) | 30.57% | 0% | Yes | Yes |
| Histological | Mononuclear cells present | Not present (0); present (1) | 30.57% | 0% | Yes | Yes |
| Histological | Sarcoplasmic inflammatory infiltrate | Not present (0); present (1) | 30.57% | 0% | Yes | Yes |
| Histological | Fibre necrosis | Not present (0); present (1) | 30.57% | 0% | Yes | Yes |
| Histological | Interstitial oedema | Not present (0); present (1) | 30.57% | 0% | Yes | Yes |

| | | | | | | |
|---|---|---|---|---|---|---|
| Histological | Increased glycogen | Not present (0); present (1) | 30.57% | 0% | Yes | Yes |
| Histological | Amylase-resistant polyglucosan | Not present (0); present (1) | 30.57% | 0% | Yes | Yes |
| Histological | Abnormal periodic anti-Schiff (PAS) staining | Not present (0); present (1) | 30.57% | 0% | Yes | Yes |
| Histological | Sarcocyst | Not present (0); present (1) | 30.57% | 0% | Yes | Yes |
| Histological | Fat infiltration | Not present (0); present (1) | 30.57% | 0% | No | No |
| Histological | Clustered adipocytes | Not present (0); present (1) | 30.57% | 0% | No | No |
| Histological | Group atrophy | Not present (0); present (1) | 32.12% | 4.14% | No | No |
| Histological | Fibre type atrophy/loss/ effect | Not present (0); present (1) | 54.29% | 32.41% | No | No |
| Histological | Fibre type grouping | Not present (0); present (1) | 54.38% | 33.10% | No | No |
| Histological | Abnormalities on oil red O staining | Not present (0); present (1) | 55.84% | 31.72% | No | No |
| Histological | Abnormal myelin | Not present (0); present (1) | 88.59% | 85.52% | No | No |
| Histological | Myelin loss | Not present (0); present (1) | 88.41% | 85.52% | No | No |
| Histological | Nerve degeneration | Not present (0); present (1) | 88.23% | 84.14% | No | No |
| Histological | Neuritis | Not present (0); present (1) | 89.05% | 88.28% | No | No |
| Histological | Lobulated fibres | Not present (0); present (1) | 57.66% | 35.17% | No | No |
| Histological | Ragged red fibres | Not present (0); present (1) | 58.39% | 35.17% | No | No |
| Histological | Rod bodies | Not present (0); present (1) | 58.39% | 35.17% | No | No |
| Histological | Tubular aggregates | Not present (0); present (1) | 58.39% | 35.17% | No | No |
| Histological | Abnormalities on Gomori trichrome staining | Not present (0); present (1) | 58.30% | 34.48% | No | No |
| Histological | Abnormalities on NADH staining | Not present (0); present (1) | 72.81% | 55.17% | No | No |

| | | | | | | |
|---|---|---|---|---|---|---|
| Histological | Abnormalities on SDH staining | Not present (0); present (1) | 72.81% | 55.17% | No | No |
| Histological | Abnormalities on COX staining | Not present (0); present (1) | 72.81% | 55.17% | No | No |
| Histological | Abnormalities on acid phosphatase staining | Not present (0); present (1) | 99.72% | 100% | No | No |
| Histological | Abnormalities on dystrophin immunohistoc-hemistry | Not present (0); present (1) | 99.91% | 100% | No | No |
| Histological | Abnormalities on Alizarin red staining | Not present (0); present (1) | 99.91% | 100% | No | No |
