# Supplementary material for: Characterisation of phenotypic patterns in equine exercise‐associated myopathies
Source: Equine Vet J. 2024 Jul 5;57(2):347–61. doi: 10.1111/evj.14128 (PMC11807944; doi:10.1111/evj.14128)
Supplement: Supplementary file 15 — Table S2. Variables scored for blinded histology study. [file EVJ-57-347-s016.pdf]

Equine Veterinary Journal BEVA

Table S2: Variables scored for blinded histology study.

**Variable**	**Data type**	**Scoring**
Fibre size variation severity	Ordinal	0-5; where 0 is not present and 5 is most severe
Fibre size variation distribution	Categorical	Diffuse, regional, or focal
Internalised nuclei severity	Ordinal	0-5; where 0 is not present and 5 is most severe
Internalised nuclei distribution	Categorical	Diffuse, regional, or focal
Centralised nuclei severity	Ordinal	0-5; where 0 is not present and 5 is most severe
Centralised nuclei distribution	Categorical	Diffuse, regional, or focal
Fibre hypertrophy severity	Ordinal	0-5; where 0 is not present and 5 is most severe
Fibre hypertrophy distribution	Categorical	Diffuse, regional, or focal
Fibre hypercontraction severity	Ordinal	0-5; where 0 is not present and 5 is most severe
Fibre hypercontraction distribution	Categorical	Diffuse, regional, or focal
Fibre atrophy severity	Ordinal	0-5; where 0 is not present and 5 is most severe
Fibre atrophy distribution	Categorical	Diffuse, regional, or focal
Fibre angular atrophy severity	Ordinal	0-5; where 0 is not present and 5 is most severe
Fibre angular atrophy distribution	Categorical	Diffuse, regional, or focal
Fibre splitting severity	Ordinal	0-5; where 0 is not present and 5 is most severe
Fibre splitting distribution	Categorical	Diffuse, regional, or focal
Whorled fibre severity	Ordinal	0-5; where 0 is not present and 5 is most severe
Whorled fibre distribution	Categorical	Diffuse, regional, or focal
Cytoplasmic body/mass severity	Ordinal	0-5; where 0 is not present and 5 is most severe
Cytoplasmic body/mass distribution	Categorical	Diffuse, regional, or focal

Subsarcolemmal vacuolation severity	Ordinal	0-5; where 0 is not present and 5 is most severe
Subsarcolemmal vacuolation distribution	Categorical	Diffuse, regional, or focal
Cytoplasmic vacuolation severity	Ordinal	0-5; where 0 is not present and 5 is most severe
Cytoplasmic vacuolation distribution	Categorical	Diffuse, regional, or focal
Rimmed vacuole severity	Ordinal	0-5; where 0 is not present and 5 is most severe
Rimmed vacuole distribution	Categorical	Diffuse, regional, or focal
Myofibrillar aggregate severity	Ordinal	0-5; where 0 is not present and 5 is most severe
Myofibrillar aggregate distribution	Categorical	Diffuse, regional, or focal
Endomyseal fibrosis severity	Ordinal	0-5; where 0 is not present and 5 is most severe
Endomyseal fibrosis distribution	Categorical	Diffuse, regional, or focal
Fibre necrosis severity	Ordinal	0-5; where 0 is not present and 5 is most severe
Fibre necrosis distribution	Categorical	Diffuse, regional, or focal
Inflammatory cellular infiltration severity	Ordinal	0-5; where 0 is not present and 5 is most severe
Inflammatory cellular infiltration distribution	Categorical	Diffuse, regional, or focal
Glycogen accumulation/depletion severity	Ordinal	1-5; where 1 is severely depleted, 3 is normal glycogen content, and 5 is most severely accumulated
Glycogen appearance and distribution	Categorical	Absent from fibres, endomyseal, granular, fine, aggregated, and/or patchy (all selected that applied)
Polyglucosan severity	Ordinal	0-5; where 0 is not present and 5 is most severe
Polyglucosan distribution	Categorical	Diffuse, regional, or focal
Sarcocyst severity	Ordinal	0-5; where 0 is not present and 5 is most severe
Sarcocyst distribution	Categorical	Diffuse, regional, or focal

Sarcoplasmic lipid accumulation severity	Ordinal	0-5; where 0 is not present and 5 is most severe
Sarcoplasmic lipid accumulation	Categorical	Diffuse, regional, or focal
Endomyseal lipid severity	Ordinal	0-5; where 0 is not present and 5 is most severe
Endomyseal lipid distribution	Categorical	Diffuse, regional, or focal
Lobulated fibre severity	Ordinal	0-5; where 0 is not present and 5 is most severe
Lobulated fibre distribution	Categorical	Diffuse, regional, or focal
Rod body severity	Ordinal	0-5; where 0 is not present and 5 is most severe
Rod body distribution	Categorical	Diffuse, regional, or focal
Tubular aggregate severity	Ordinal	0-5; where 0 is not present and 5 is most severe
Tubular aggregate distribution	Categorical	Diffuse, regional, or focal
Desmin aggregate severity	Ordinal	0-5; where 0 is not present and 5 is most severe
Desmin aggregate distribution	Categorical	Diffuse, regional, or focal
Desmin staining description	Categorical	Fingerprint-like deposits, occasional dense staining, diffuse staining, sarcolemmal staining, dense-staining atrophied fibres, fibre caps, occasional accumulation, central accumulation (all selected that applied)
SDH/COX/NADH description	Categorical	Pale staining, core-like lesions, 'moth-eaten' fibres, small oxidative fibres, oxidative atrophy, loss of chequer board pattern (all selected that applied)
Artefact description	Categorical	Freeze artefact, saline artefact, other artefact (all selected that applied)
Other features	Categorical	Polyglucosan in nerve, Globoid nuclei, generalised atrophy, fibre caps, generalised separation, centronuclear appearance (all selected that applied)
