# Supplementary material for: Characterisation of phenotypic patterns in equine exercise‐associated myopathies
Source: Equine Vet J. 2024 Jul 5;57(2):347–61. doi: 10.1111/evj.14128 (PMC11807944; doi:10.1111/evj.14128)
Supplement: Supplementary file 16 — Table S3. Significance of longlist variables between k‐means phenotypic subtypes, and between classic RER and non‐classic EAMS subtype. [file EVJ-57-347-s006.pdf]

Table S3: Significance of longlist variables between k-means phenotypic subtypes, and between classic RER and non-classic EAMS subtype.

Variable type	Variable	Associated subtype	p-value	
			4 phenotypic subtypes	Classic RER vs non-classic EAMS
Histological	Whorled fibres	3	**<0.001***	0.620
Clinical	Abnormal gait	4	**<0.001***	**<0.001***
Histological	Inclusions in myofibres	2	**<0.001***	0.916
Histological	Myofibrillar separation and/or disruption	3	**<0.001***	0.620
Clinical	Muscle pain	4	**<0.001***	**<0.001***
Histological	Fibre vacuolation	3	**0.006#**	0.426
Clinical	Shivers	2	**0.044#**	0.358
Clinical	Weakness	4	**0.017#**	**0.001***
Clinical	Ataxia	4	**0.017#**	**0.001***
Clinical	Reluctance to go forward	4	**0.019#**	**0.001***
Clinical	Abnormal head carriage	4	0.060	**0.020#**
Histological	Necrotic fibres	-	0.081	0.060
Signalment	Cob breed type	-	0.075	0.992
Histological	Fibre size variation	-	0.725	0.343
Histological	Fibre hypertrophy	-	0.239	0.523
Histological	Increased intracellular glycogen	-	0.248	0.577
Signalment	Irish breed type	-	0.361	0.347
Clinical	Muscle atrophy	-	0.136	0.059
Clinical	Muscle oedema	-	0.136	0.059
Histological	Diastase-resistant PAS-positive deposits	-	0.136	0.059
Signalment	Age	-	0.636	0.742

Histological	Hypercontracted fibres	-	0.349	0.260
Clinical	Recurrent ER episodes	-	0.074	0.083
Clinical	Myoglobinuria	-	0.283	0.814
Signalment	Warmblood breed type	-	0.394	0.355
Histological	Abnormalities on PAS staining	-	0.630	0.727
Clinical	Poor performance	-	0.553	0.478
Histological	Interstitial fibrosis	-	0.558	0.657
Histological	Internalised nuclei	-	0.650	0.628
Clinical	Stiffness	-	0.969	0.875
Histological	Histological severity	-	0.499	0.597
Signalment	Sex	-	0.854	0.589
Histological	Histological disease stage	-	0.810	0.460
Histological	Fibre atrophy	-	0.949	0.992

*: denotes a p-value that is significant at a Bonferroni-corrected threshold of 0.0015; $^{\#}$: denotes a p-value that is significant at a nominal p of 0.05. Shortlisted variables for further study are in bold. The classic RER subtype was the same as phenotypic subtype 1 (n=87), whilst the non-classic EAMS subtype consisted of phenotypic subtypes 2, 3 and 4 (n=22).
