# Supplementary material for: Characterisation of phenotypic patterns in equine exercise‐associated myopathies
Source: Equine Vet J. 2024 Jul 5;57(2):347–61. doi: 10.1111/evj.14128 (PMC11807944; doi:10.1111/evj.14128)
Supplement: Supplementary file 17 — Table S4. Significance of longlist variables between k‐means phenotypic subtypes, and between classic RER and non‐classic EAMS subtypes, in Set 1 and Set 2. [file EVJ-57-347-s002.pdf]

Equine Veterinary Journal BEVA

Table S4: Significance of longlist variables between k-means phenotypic subtypes, and between classic RER and non-classic EAMS subtypes, in Set 1 and Set 2.

**Variable**	**p-value**			
	**Set 1**		**Set 2**	
	**4 phenotypic subtypes**	**Classic RER vs non-classic EAMS subtypes**	**5 phenotypic subtypes**	**Classic RER vs non-classic EAMS subtypes**
Hepatopathy or hepatitis			**<0.0001**[*]	2.03E-01
Poor performance			**<0.0001**[*]	**<0.0001**[*]
Abnormal gait	**<0.0001**[*]	**<0.0001**[*]	**<0.0001**[*]	**<0.0001**[*]
Stiffness			**<0.0001**[*]	**<0.0001**[*]
Recurrent ER episodes			**<0.0001**[*]	**<0.0001**[*]
Muscle pain	**<0.0001**[*]	**<0.0001**[*]	**<0.0001**[*]	**2.66E-04**[*]
Ataxia	**<0.0001**[*]	**<0.0001**[*]	**3.34E-04**[*]	**6.47E-03**[#]
Crossbreed			9.83E-02	8.58E-01
Behavioural changes			**5.33E-04**[*]	1.06E-01
Other breed			8.66E-02	5.04E-01
Age			8.55E-01	1.65E-01
Shivers	5.61E-02	6.55E-02	**5.17E-03**[#]	3.71E-01
Lethargy			**1.85E-04**[*]	**4.46E-02**[#]
Thoroughbred breed			2.04E-01	**4.66E-02**[#]
Cob breed			3.41E-01	1.11E-01
Warmblood breed			9.99E-01	7.87E-01
Male sex			4.07E-01	1.08E-01
Reluctance to go forward	**4.83E-02**[#]	**1.13E-02**[#]	4.51E-01	1.73E-01
Abnormal head carriage	8.69E-02	1.94E-01		
Inclusions	9.29E-01	3.93E-01		
Fibre vacuolation	**1.29E-02**[#]	3.15E-01		
Whorled fibres	**<0.0001**[*]	**4.27E-03**[*]		
Myofibrillar separation and/or disruption	**<0.0001**[*]	**2.85E-02**[#]		
Weakness	**<0.0001**[*]	**<0.0001**[*]		

*: denotes a p-value that is significant at a Bonferroni-corrected threshold of 0.0015; [#]: denotes a p-value that is suggestive at a nominal p of 0.05. Shortlisted variables for further study are in bold. The classic RER subtype was the same as phenotypic subtype 2 in both Sets, whilst in Set 1 the non-classic EAMS subtype consisted of phenotypic subtypes 1, 3 and 4, and in Set 2 consisted of phenotypic subtypes 1, 3, 4 and 5.
