# Supplementary material for: Characterisation of phenotypic patterns in equine exercise‐associated myopathies
Source: Equine Vet J. 2024 Jul 5;57(2):347–61. doi: 10.1111/evj.14128 (PMC11807944; doi:10.1111/evj.14128)
Supplement: Supplementary file 18 — Table S5. Correlation between histological variable scores and desmin aggregate score. [file EVJ-57-347-s005.pdf]

Equine Veterinary Journal BEVA

Table S5: Correlation between histological variable scores and desmin aggregate score.

**Variable 1**	**Variable 2**	**Spearman's ρ**	**p-value**
Desmin aggregate score	Myofibrillar aggregate score	-0.060	0.626
Desmin aggregate score	Freeze artefact	**-0.320**	**0.008**
Desmin aggregate score	Saline artefact	-0.003	0.980
Desmin aggregate score	Myofibrillar loss/separation score	-0.139	0.258
Desmin aggregate score	Fibre necrosis score	**-0.317**	**0.009**
Desmin aggregate score	Fibre size variation score	0.037	0.766
Desmin aggregate score	Internalised nuclei score	0.201	0.100
